# A comparison of genetic connectivity in two deep sea corals to examine whether seamounts are isolated islands or stepping stones for dispersal

**DOI:** 10.1038/srep46103

**Published:** 2017-04-10

**Authors:** Karen J. Miller, Rasanthi M. Gunasekera

**Affiliations:** 1 Australian Institute of Marine Science, Indian Ocean Marine Research Centre, The University of Western Australia (MO96), 35 Stirling Hwy, Crawley, Western Australia 6009, Australia; 2 CSIRO Oceans & Atmosphere Flagship, GPO Box 1538, Hobart, Tasmania 7001, Australia.

## Abstract

Ecological processes in the deep sea are poorly understood due to the logistical constraints of sampling thousands of metres below the ocean’s surface and remote from most land masses. Under such circumstances, genetic data provides unparalleled insight into biological and ecological relationships. We use microsatellite DNA to compare the population structure, reproductive mode and dispersal capacity in two deep sea corals from seamounts in the Southern Ocean. The solitary coral *Desmophyllum dianthus* has widespread dispersal consistent with its global distribution and resilience to disturbance. In contrast, for the matrix-forming colonial coral *Solenosmilia variabilis* asexual reproduction is important and the dispersal of sexually produced larvae is negligible, resulting in isolated populations. Interestingly, despite the recognised impacts of fishing on seamount communities, genetic diversity on fished and unfished seamounts was similar for both species, suggesting that evolutionary resilience remains despite reductions in biomass. Our results provide empirical evidence that a group of seamounts can function either as isolated islands or stepping stones for dispersal for different taxa. Furthermore different strategies will be required to protect the two sympatric corals and consequently the recently declared marine reserves in this region may function as a network for *D. dianthus,* but not for *S. variabilis*.

Despite recent developments in ocean observing and exploration, the deep sea remains one of the last scientific frontiers. The marine communities that exist at depths in excess of 1000 m are some of the least understood ecosystems on the planet, but they face considerable threats from fishing, mining and climate change and there is an urgency to document their diversity and understand threatening processes to provide a foundation for effective management and conservation of deep sea resources^1^,^2^,^3^.

Evidence of anthropogenic-induced declines in deep sea populations is growing. Fishing, particularly on seamounts, has resulted in depletion of many fish stocks, as well as having indirect effects on benthic communities including ecosystem engineers such as sponges and corals that are very slow to recover^4^,^5^,^6^. Population declines can result in reduced genetic diversity and resilience, hence ensuring connectivity among populations is integral to recovery of exploited populations and future conservation of these vulnerable marine ecosystems^7^. Accordingly, research on connectivity, particularly in deep sea ecosystems, must be prioritised for future management needs^1^.

For many marine species, connectivity is maintained through the planktonic dispersal of microscopic larvae or juveniles, and an assumption of a direct link between larval duration and dispersal distance has formed the basis for predicting connectivity in poorly studied ecosystems such as the deep sea^8^,^9^. Increasingly, however, studies are revealing such paradigms are not widely upheld in marine systems, and that many factors will collectively influence realised dispersal^10^,^11^. These include hydrodynamic features such as oceanic fronts, upwellings, and the stratification of water masses, all of which can constrain the dispersal of long-lived larvae^12^,^13^,^14^,^15^. In contrast, rafting can result in long-range dispersal for species that have direct-developing larvae with no dispersal phase^16^ and larval behaviours can facilitate dispersal in currents^17^,^18^. Even anthropogenic factors such as pollution and coastal runoff can affect dispersal and population genetic structure^19^ and isolation-by-distance, although intuitively satisfying as a model for larval dispersal, is only rarely found in marine populations with planktonic larvae e.g. ref. 20.

The mismatch between expected and observed dispersal of marine invertebrates has highlighted the need to incorporate environmental parameters with biological, ecological and genetic data to parametrise models that better explain population or metapopulation structure and predict dispersal in the ocean; hence a growing focus on “seascape genetics” in studies of connectivity in the sea^21^,^22^,^23^,^24^,^25^. Even so, there exist limitations to understanding dispersal at depths >1000 m due to a paucity of data on deep ocean currents and the biological and environmental conditions that might affect dispersal including the reproductive mode and timing of most deep sea species^26^,^27^. Moreover, despite a burgeoning literature on deep sea genetic connectivity, studies are geographically disparate, taxonomically diverse, plagued by low sample sizes, and poor in spatial and temporal replication^1^,^28^ making it hard to draw general conclusions^29^.

Notably, most deep sea genetic studies have focussed on large-scale patterns of dispersal across thousands of kilometres between geographic features such as oceanic ridges or at continental scales e.g. refs 15, 30, 31, 32, 33, 34, and there have been few studies of small-scale (m to km) patterns of connectivity in the deep sea. In large part this is due to the logistical difficulties of sampling at depth, resulting in fewer samples per site, and confounded by low resolution of genetic markers used^28^. Increasingly, however, shallow water studies are showing that patchiness in population genetic structure usually exists at small scales, and that for many species, dispersal may be limited to just a few hundred metres or kilometres^35^,^36^,^37^,^38^. Thus to fully understand ecological relationships in deep sea communities it will be important to study processes occurring both at small, as well as large, spatial scales. Of the few deep-sea studies that have examined small spatial scales, there is evidence that patterns similar to those seen in shallow water may exist. For example, Shank & Halanych^39^ were able to distinguish among populations and cohorts of the vent tubeworm *Riftia pachyptila,* and reported genetic structure across distances as little as 400 m. Baco & Shank^40^ found genetic differentiation among some seamount populations of the gorgonian *Corallium lauuense* in Hawaii. Similarly, genetic studies of the deep sea coral *Lophelia pertusa* have revealed evidence of limited dispersal and genetic differentiation among some populations at scales of tens of kilometres^41^.

Here we combine population genetic data based on microsatellite genotypes of two sympatric and common deep sea coral species - the solitary coral *Desmophyllum dianthus* and the matrix-forming, colonial coral *Solenosmilia variabilis* - to predict dispersal within and among seamount populations in the Southern Ocean. These two species of deep sea coral belong to the scleractinian family Caryophyllidae and are abundant and widely distributed on seamounts in the Southern Ocean. The solitary coral, *Desmophyllum dianthus*, has been recorded to depths of almost 2400 m^42^ and occurs throughout the world’s oceans. Genetic studies of ITS, 16S, and the mitochondrial control region have shown that populations of *D. dianthus* on widely separated ridge systems are isolated (1000s of km^30^) and there is evidence that oceanographic features such as interfaces between vertically stratified water masses within the Southern Ocean are barriers to vertical dispersal in this species^15^. Unfortunately little is known about the early life history of *D. dianthus*, but it is presumed to be a seasonal gonochoric spawner like most other species within the family^43^,^44^ and is therefore likely to have a relatively short (e.g. days-weeks) planktonic dispersal phase.

The branching coral *Solensomilia variabilis* has been likened to the Southern Ocean equivalent of *Lophelia pertusa* both structurally and ecologically. It forms extensive beds on shelf and seamount structures and is an important habitat forming species. In contrast to *D. dianthus*, DNA sequence data of the 16 S and mitochondrial control regions showed no evidence of subdivision among populations of *S. variabilis* from southern Australia and New Zealand^30^ which may reflect long-distance dispersal of larvae, or this may be an artefact of the conserved nature of mtDNA in anthozoans^45^. Like most deep sea corals, we also know little about reproductive mode and timing in *S. variabilis*, although mature gametes have been observed in New Zealand specimens in April^44^, confirming that it is gonochoric and most likely broadcast spawns in late April or May (Autumn) in the Southern Ocean, although reproduction in *S. variabilis* in the South Atlantic may be continuous^46^. *Solenosmilia variabilis* likely undergoes asexual reproduction based on observations of intratentacular budding^44^.

Here we have sampled both species across the same spatial scales, from oceanic ridges to multiple sites on a single seamount (Fig. 1) in order to make direct comparisons of the scale of dispersal and better understand ecological and evolutionary processes in these deep seamount ecosystems. In addition, recognising the increasing exploitation of deep sea ecosystems, we were able to compare genetic diversity and structure between fished and unfished seamounts to determine if there was evidence that fishing activities had reduced resilience of seamount coral communities.

## Results

### Genetic diversity in deep sea corals

Genetic diversity in *D. dianthus* from nine seamounts in the Southern Ocean was high. Across the nine microsatellite loci genotyped in this species, we recorded between 13 and 31 alleles, with an average of 21.25 alleles per locus overall, and an average of 8.3 alleles per locus within each of the 16 sampling sites (Table S4). Of the 326 *D. dianthus* genotyped, there were 324 unique genotypes. Only two genotypes occurred more than once in the sample; two corals from Macquarie Ridge Seamount 5 Station 50 shared the same 8-locus genotype and two individuals from Hill Z56 in the Tasmanian Seamounts shared the same 8-locus genotype. The probability that each of these repeat genotypes arose more than once in the sample through sexual reproduction was incredibly low (<5.75 × 10^−11^) and so it is likely these are the result of asexual reproduction.

Heterozygosity observed in *D. dianthus* populations ranged from 0.438 to 0.778 and was lower than expected in all populations (Table S4). Of the eighty single-locus x site tests for Hardy-Weinberg equilibrium in *D. dianthus* (for those sites where n > 12), more than half (52%) of these were significant, with all representing heterozygote deficits. This pattern did not change after data were adjusted for the presence of null alleles.

Genetic diversity in *S. variabilis* was lower than that observed in *D. dianthus*. We recorded between 3 and 34 alleles per locus across the nine seamounts (average = 15.2), and an average of 5.4 alleles per locus at each of the 18 sites (Table S5). Observed heterozygosity ranged from 0.335 to 0.529 and was lower in all populations than expected (Table S5). There were 85 instances of departures from HWE in 134 tests, and 71 of these represented heterozygote deficits. These were in part related to clonality within the populations. Of the 589 samples genotyped, there were only 327 unique genotypes of which 83 were represented by more than one sample. Some of these were quite common (represented by 10–15 individuals) and specimens that shared genotypes were usually collected from the same site. Four clonal genotypes were recorded from two different sites; with two of these being different sites on the same seamount. The probability that repeat genotypes arose through sexual reproduction was low (<1.2 × 10^−4^) hence they most likely represent the product of asexual reproduction. Estimates of genotypic richness in *S. variabilis* averaged 0.56 across all sites, but ranged from 0.22 to 0.88 across the nine seamounts (Table 1) indicating varying levels of asexual reproduction at different sites. Clonal genotypes were subsequently removed from the *S. variabilis* data set and estimates of HWE recalculated. This decreased the number of departures from HWE to just 32 of 134 tests (28 of which were heterozygote deficits), but most of these were associated with Locus *SvD213* which had large heterozygote deficits at 14 of the 18 sites sampled and was likely to have null alleles. Excluding *SvD213*, and with only 18 of the remaining 116 locus x site tests being significant, we considered the sexual populations of *S. variabilis* at each site to be in Hardy Weinberg Equilibrium.

Despite the known impacts of deep sea fishing on Tasmanian seamounts^47^,^48^, there was no evidence that fishing activity had reduced genetic diversity in the coral populations. Comparisons of allelic richness and expected heterozygosity revealed no significant differences in diversity between fished and unfished seamounts either for *D. dianthus* or *S. variabilis* (Table 2). Furthermore private alleles were evident in most populations of both species. For *D. dianthus* multiple private alleles were detected at all but three of the 16 sampling sites and with an average of 2.8 (±0.65SE) private alleles/site. For *S. variabilis*, there were private alleles recorded at 16 of the 17 sites studied, with an average of 2.2 (±0.39SE) private alleles/site.

### Spatial genetic structure within species

There was evidence of multiple genetic groups within each of the two deep sea coral species. For *D. dianthus* STRUCTURE analysis suggested K = 2 (based on ΔK) (Figs 2a and S1a) or K = 3 (based on L(K)) (Figs 2b and S1b). For K = 3 there was a clear link between genetic structure and depth (Fig. 2), which agrees with findings based on DNA sequence data^15^ that depth is an important structuring force in this coral species. Principal coordinates analysis also revealed clear groupings of sites based on depth rather than geographic location (Fig. 3) and provided further support for the existence of three depth-related groups based on microsatellite data. Additional STRUCTURE runs on the mid-depth cluster and mid+deep cluster did not reveal any evidence of sub-structure linked to geographic location (i.e. seamount).

For *S. variabilis*, the number of populations within the data set was also K = 2 (based on ΔK) (Figs 4a and S2a) or K = 3 (based on L(K)) (Figs 4b and S2b). However, in contrast to *D. dianthus*, this structure exists within a single depth stratum (mid-depth 1000–1400 m). Interestingly, while some sites appear to be a mix of the 2 (or 3) genetic groups (e.g. HillU Sites 13 & 14, Little Mongrel) other sites are dominated by a single type (e.g. Hill U Sites 12 & 15, Tas 1200, Mini Matt Site 34) (Fig. 4a,b), although there was no clear relationship evident between genetics and location based on principal coordinates analysis (results not shown).

For *D. dianthus* it was not possible to test for oceanographic-scale genetic structure as replication was confounded by the depth stratification. However for S. *variabilis* there was significant genetic differentiation between the Macquarie Ridge and all other sites (pairwise *F*_*ST*_ ranged from 0.044–0.162, p < 0.001 across all comparisons). This is quite different to the findings based on DNA sequence data (16 S and control region) which revealed a single common haplotype in *S. variabilis* populations spanning Western Australia, Tasmania, Macquarie Ridge and New Zealand^30^, reinforcing that microsatellite markers are better suited to revealing population structure than mtDNA sequences for corals^45^.

### Gene flow among seamounts within a single depth stratum (1000–1400 m)

Within the 1000–1400 m depth stratum on Tasmanian Seamounts, the two closely related deep sea corals have very different population genetic structure and gene flow. For *D. dianthus* there are only very low levels of genetic subdivision, with two of three test statistics suggesting gene flow occurs among the seamounts (*F*_*ST*_ = 0.009, p < 0.001; *G*’_*ST*_ = 0.036, p = 0.128; *D*_*EST*_ = 0.03, p = 0.129 considering all sites; *F*_*ST*_ = 0.006, p < 0.001; *G*’_*ST*_ = 0.024, p = 0.061; *D*_*EST*_ = 0.021, p = 0.062 across sites where n > 12). Pairwise comparisons showed significant differences between sites in only one comparison, between Hill Z15 and Dory Hill and again this was significant based on *F*_*ST*_, but not *D*_*EST*_ (Table 3). Estimates of gene flow for *D. dianthus* were moderate, with *N*_*e*_*m* = 43.6 (based on *F*_*ST*_), *N*_*e*_*m* = 5.6 (based on private alleles) and *N*_*e*_*m* = 19.6 based on coalescent analyses.

For *S. variabilis* there is significant genetic subdivision among all the Tasmanian seamounts (*F*_*ST*_ = 0.111, p < 0.001; *G*’_*ST*_ = 0.167, p < 0.001; *D*_*EST*_ = 0.103, p < 0.001) indicating populations are not panmictic. Most pairwise comparisons among seamounts showed significant genetic differentiation indicative of limited gene flow, with the exception of four of the 153 tests (between L Mongrel and Mini Matt Stn 34, Dory Hill and Tasman 1200, Dory Hill and Mini Matt Stn 33 and Hill Z56 and Mini Matt Stn 33; Table 4). Estimates of gene flow were considerably lower in *S. variabilis* than in *D. dianthus*, with *N*_*e*_*m* = 3.752 (based on *F*_*ST*_), *N*_*e*_*m* = 2.25 (based on private alleles) and *N*_*e*_*m* = 0.94 based on coalescent analyses.

There was no evidence of isolation by distance in either species across the Tasmanian Seamounts (*D. dianthus*: Mantel’s R^2^ = 0.025, p = 0.321; *S. variabilis* Mantel’s R^2^ = 0.014, p = 0.106). For *D. dianthus*, values of *N*_*e*_*m* based on coalescent estimates of migration were generally large and ranged from 0.09 to 156. Of the 50 estimates of migration between all pairs of sites, 38 resulted in *N*_*e*_*m* > 1, and 20 of these with *N*_*e*_*m* > 10 indicating sufficient gene flow for ecological connectivity among most seamounts^49^. Seventy three percent of pairwise estimates of *N*_*e*_*m* among seamounts were significantly greater than 1 (and only 3% significantly <1; Table S6) emphasising the considerable gene flow among populations of *D. dianthus*. Furthermore, in 15 of the 25 seamount pair comparisons there was significant bi-directionality in migration (Fig. 5, Table S6) and overall ≈70% of migration in *D. dianthus* occurred in a westerly direction. This is consistent with estimates of particle dispersal at ~1000 m in the Tasmanian Seamount region which predicts a westward flow in the months when *D. dianthus* is most likely to be spawning (Fig. 6). However, tests of four different models of gene flow showed strong support only for a full model of dispersal (p > 0.9) above a westward, eastward or panmictic pattern of dispersal, suggesting either that reproduction and dispersal occurs outside of the period of predominately westward flow or other mechanisms are influencing gene flow in this species.

For *S. variabilis*, pairwise estimates of *N*_*e*_*m* based on coalescent estimates of migration among seamounts were low and ranged from 0.001 to 10.76 indicating insufficient gene flow between any seamounts for ecological connectivity. In fact 49 of the 56 estimates were <1 indicating that seamount populations of *S. variabilis* are ecologically isolated and only 5% of estimates showed *N*_*e*_*m* significantly greater than one (Table S7). For *S. variabilis* nine of the 28 pairwise estimates of *N*_*e*_*m* were significantly different to each other, although since most of these involved *N*_*e*_*m* < 1, are likely meaningless in the context of directional gene flow. Notably the highest estimated gene flow occurred into Hill Z15 from Hill Z56 (*N*_*e*_*m* = 10.75; eastward flow) compared with *N*_*e*_*m* of 0.49 in the opposite direction and into MiniMatt from Hill Z56 (*N*_*e*_*m* = 9.35; westward flow) compared with *N*_*e*_*m* of 2.84 in the opposite direction.

Estimates of effective population size also differed between the two coral species, being much smaller in *S. variabilis* (mean *Θ* = 1.31; 95% CI 1.19–1.47) than in *D. dianthus* (mean *Θ* = 9.81; 95% CI 7.61–10.01). Contemporary estimates of effective population size revealed similar patterns, with populations of *S. variabilis* relatively small but more variable across sites than *Θ* (mean N_e_ = 11.69; 95% CI 6.65–68.7) and populations of *D. dianthus* very large (mean N_e_ = infinite; although this likely reflects limitations of calculating N_e_ for populations where N_e_ is >1000, rather than an accurate estimate of effective population size^50^).

### Gene flow within seamounts

The genetic structure of coral populations among sites within the same depth stratum on a seamount was very different for *D. dianthus* and *S. variabilis*. There was small-scale structure in *S. variabilis* both at Hill U and Mini Matt, with pairwise comparisons (based on *F*_*ST*_ and Jost’s D) significantly different to zero in most comparisons (Table 4) suggesting limited gene flow among sites even separated by as little as a few hundred metres on the same seamount. Different genetic populations among the sites at Hill U are also apparent from the STRUCTRUE analysis (Fig. 4a,b). The structuring in *S. variabilis* is the opposite of what we found at mid-depth for *D. dianthus*, although unfortunately sample sizes were less robust at the within-seamount scale in this species and consequently the results are less clear. On Hill U, there was no significant genetic differentiation between Stations 13 and 14 which are separated by approximately 700 m based on *G*’_*ST*_ (0.005, p = 0.217) and Jost’s *D* (0.021, p = 0.229) although *F*_*ST*_ was significant (0.011, p < 0.05). Similarly on the Macquarie Ridge, the two shallow sites sampled on the top of Seamount 5 (~3 km apart) were not significantly differentiated from each other based on *G*’_*ST*_ (0.003, p = 0.229) and Jost’s *D* (0.021, p = 0.242) but were based on *F*_*ST*_ (=0.016, p < 0.05) and the same pattern was evident for the three deep sites sampled on the Cascade Plateau and that are separated by as little as 300 m. However the consensus between *G*’_*ST*_ and Jost’s *D* is compelling evidence^51^ that there is no genetic differentiation among *D. dianthus* populations at the within-seamount scale, and that contemporary gene flow occurs among these sites. Coalescent estimates of migration among sites are, however, low (Table S6) and in line with significant *F*_*ST*_ results found in comparisons among sites both within and among seamounts for *D. dianthus* which likely reflects historical structure among populations.

Within seamounts there was little evidence of directional migration among sites within the same depth stratum. For *D. dianthus*, coalescent estimates of m and *N*_*e*_*m* indicated similar levels of exchange in either direction among sites on Hill U, among sites on Cascade Plateau and between sites on Seamount 5 on the Macquarie Ridge (Table S8), the only exception being that there was almost ten times more migration from Station 15 to Station 14 on Hill U than in the opposite direction (net north-westerly flow). Furthermore, most estimates of *N*_*e*_*m* were significantly greater than one (Table S8). Overall these results suggest *D. dianthus* populations on seamounts are well mixed, at least within depth zones.

For *S. variabilis*, there was also limited evidence of directional migration among sites within seamounts, particularly at Mini Matt, noting that all estimates of m and *N*_*e*_*m* are very low, with many being significantly less than one indicating populations are effectively isolated (Table S9). However on Hill U there was some indication that Stations 12 and 13 were a source of migrants to Stations 14, 15 and 16, although this resulted in little effective gene flow among sites as *N*_*e*_*m* was <10 in all instances (Table S9).

## Discussion

Seamounts have variously been considered as isolated islands of endemicity or stepping stones for dispersal across ocean divides^52^; here we provide genetic evidence that both hypotheses are valid. Data from hypervariable microsatellite DNA markers have shown the solitary coral *Desmophyllum dianthus* has relatively large effective population sizes and considerable gene flow indicating the seamounts are acting as stepping stones for connectivity among sites in the Tasmanian Seamount Region at depths of 1000–1400 m. In contrast, the matrix-forming *Solenosmilia variabilis* across the same set of seamounts and depth range has small effective population size, populations are genetically differentiated, asexual reproduction appears to be an important aspect of its life history, and there is limited gene flow and larval dispersal even on relatively small spatial scales among sites on a single seamount. The starkly contrasting nature of these coral populations provides considerable insight into the variety of ecological processes operating in the deep sea.

### Links between ocean currents, distribution patterns and larval dispersal in deep sea corals

The distinct genetic structures and contrasting connectivity in *D. dianthus* and *S. variabilis* are remarkably consistent with the species’ ranges. *D. dianthus* is common across all oceans^53^,^54^. The cosmopolitan distribution of many marine species has been used to infer widespread dispersal^55^ and this is supported in *D. dianthus* by genetic studies that reveal shared nuclear (ITS and 28S) and mitochondrial (16S and CO1) haplotypes in specimens from Australia to the Mediterranean (Addamo *et al*. 2012). Our microsatellite genetic data provides further evidence that larval dispersal and gene flow can occur in this species across hundreds of kilometres. Indeed patterns of migration appear to be influenced, at least in part, by the prevailing westerly currents across the Tasmanian Seamounts suggesting passive larval dispersal facilitated by stepping-stones such as intermediate seamounts may play a role in the distribution of this widespread and common species.

*Solenosmilia variabilis* is common in the southern hemisphere, forming extensive reef-like structures particularly around southern Australia and New Zealand^47^,^56^. It has been reported from the North Pacific and North Atlantic but is rare in these locations (www.gbif.org). A much reduced dispersal capacity, as inferred from genetic differentiation within and among Tasmanian seamounts, may explain the restricted distribution and localised abundance of *S. variabilis.* For the confamilial and matrix-forming *Lophelia pertusa*, planulae are active swimmers and can survive for at least eight weeks in the water column, although they will commence settlement behaviour after 3–5 weeks^57^. These behaviours suggest potential for long-distance dispersal, although genetic structure among reefs indicates *L. pertusa* populations are not panmictic and that dispersal may be affected by local hydrodynamic and geographic features^41^. Arguably, *S. variabils* and *L. pertusa* are the southern and northern hemisphere ecological equivalents of each other, and it is interesting that genetic structure indicative of restricted larval dispersal is apparent in both these matrix-forming species that have apparently constrained distributions.

Unfortunately too little is known about reproduction and the early life history of *D. dianthus* and *S. variabilis* to know if the approximately three times greater level of genetic differentiation seen in *S. variabilis* compared with *D. dianthus* (based on G’st and Jost’s *D*) links directly to larval duration, other aspects of their life history, or environment. For example, spawning in winter compared with summer may result in very different dispersal potential based on the magnitude of currents at 995 m in the Tasmanian Seamounts (Figure S3). Furthermore strong support of a full model of gene flow reflecting different levels of dispersal among all sites, in contrast to the more simplistic model of westerly dispersal in *D. dianthus* suggests either a different reproductive period than assumed here (January to May), or that other factors such as larval behaviour are influencing dispersal patterns. Of note also is that the hydrodynamic model used here (Figs 6 and S3) only considers processes at large scales (~10 km grid). At smaller within-seamount scales, other processes associated with boundary currents, eddies and larval behaviour will affect the dispersal of larvae, and this is evident in the variation in migration estimates among sites even within seamounts (Tables S4 and S5). Clearly more information on the timing and nature of reproduction in will be required to be able to refine oceanographic predictions and fully understand the links between currents, larval behaviour and dispersal for these deep sea coral species.

One final consideration is that the contrasting genetic structure in *D. dianthus* and *S. variabilis* could be linked to different reproductive modes. We know that *S. variabilis* is gonochoric and probably a broadcast spawner; but nothing is known of reproductive mode in *D. dianthus.* Conceivably, *D. dianthus* may brood larvae that are well provisioned and better suited to long-distance dispersal^58^. Recruitment success in deep sea gorgonians has been linked to differences between brooding and spawning life histories^59^, although notably the broadcast spawning species was more successful than the brooding species, possibly linked to larval supply rather than dispersal. However in shallow water corals, there is little evidence that brooders will be more widely dispersed or more successful than spawners^60^,^61^ or that there will be a predictable link between life history and dispersal^62^, hence reproductive mode (brooding vs. spawning) is an unsatisfactory explanation for the differences in gene flow between *D. dianthus* and *S. variabilis*.

### The importance of asexual reproduction in maintaining deep sea coral populations

We found evidence that asexual reproduction is important for localised recruitment in *S. variabilis*. This finding suggests even closer affinity with its northern hemisphere ecological equivalent *L. pertusa* which also recruits through a combination of sexual and asexual reproduction^41^,^63^,^64^. On some reefs, clonal reproduction can account for more than 50% of recruitment in *L. pertusa*^41^ and in *S. variabilis* we found as many as 76% of samples were clonal, although clearly there is variation in the importance of asexual reproduction in both species (Table 1 and refs 41, 63, 65). One concern in this study was our power to detect clonality due to the indirect method of sampling using benthic sleds which will invariably result in some fragmentation of colonies. However, with this in mind, we targeted our genetic collections at each site so as to sample progressively through each sled sample to avoid colony fragments (assuming fragments would be grouped together as a colony was crushed in the net) and in an attempt to obtain accurate estimates of genetic diversity across the sampling site. Future targeted ROV collections would be required to precisely estimate the extent and nature of clonality in *S. variabilis* i.e. through natural fragmentation, colony extension or the production of asexual larvae. However, given *S. variabilis* has been reported to reproduce asexually^44^, forms extensive mono-specific stands on seamounts^47^,^56^ and that each of our sleds likely covered tens to hundreds of metres of ground, we expect that asexual reproduction is an important life history strategy in this species. In contrast, sexual reproduction is the main source of recruitment in *D. dianthus*, further emphasising the variation of life histories even in closely related deep sea corals.

### Effects of deep-sea fishing on seamount coral populations

Although there may be evidence of reduced biomass of corals on seamounts that have been fished^4^,^5^,^48^ we found no evidence that corals on fished seamounts had lower genetic diversity than those on unfished seamounts. The loss of genetic diversity may be buffered in species with long generation times^66^, and this may explain why genetic diversity in these long-lived coral species on the Tasmanian seamounts does not appear to have been reduced to a level likely to affect their evolutionary resilience.

Our genetic data, which gives insight into life history processes in these little-understood corals, may help explain the observed effects and limited recovery of some deep sea corals following fishing. For example, *S. variabilis* populations on Tasmanian seamounts showed almost no signs of recovery 5–10 years after trawling ceased^48^. This slow recovery matches with our discovery of the importance of asexual reproduction for local recruitment in this species, as well as the limited capacity for larval dispersal and importance of local recruitment. In combination, these life history characters would certainly hinder recovery of populations where adult biomass was severely reduced.

Solitary corals are among the species that seem least affected by fishing^5^, and may also represent early colonisers on seamounts that have been impacted by fishing. Again our genetic data, which suggests a greater capacity for larval dispersal in the solitary *D. dianthus* (compared with *S. variabilis*), is consistent with their reported relative capacity to recover and/or recolonise following disturbance, and supports the suggestion that solitary corals such as *D. dianthus* will be less vulnerable to fishing effects than colonial species^5^. Importantly, it is worth noting that although the capacity for dispersal and gene flow is higher in *D. dianthus* than in *S. variabilis*, migration rates are still low to moderate in both species, and arguably lower than expected if populations were panmictic. Although only “one migrant per generation” is considered sufficient gene flow to prevent evolutionary divergence among populations, from a conservation and management perspective, *N*_*e*_*m* of >10 migrants per generation may be a more realistic measure of connectivity^49^,^67^, which is apparent at most scales for *D. dianthus*, but not for *S. variabilis*. In this context, *S. variabilis* is likely to be more vulnerable to anthropogenic impacts than *D. dianthus*.

### Implications for high seas management and conservation

Collectively, the results from this study provide critical input to the future management of deep sea corals. Based on genetic data it is clear that these two closely related coral species have very different population structures and capacity for dispersal. In this context, conservation measures such as Marine Protected Areas will need to consider the different scales across which dispersal processes are operating in order to achieve adequate protection or ensure the provision of networks of connectivity for all taxa. For example, the recently declared Commonwealth Marine Reserves in SE Australia will most likely be operating as networks for *D. dianthus*, but not for *S. variabilis*. Similarly, the size and timing of management actions such as fishing closures would have different consequences for recovery in each species. Given the importance placed on conservation of ecosystem engineers in managing marine ecosystems more broadly^68^,^69^ then clearly any measures that capture the breadth of life histories and connectivity for these two corals will undoubtedly have consequences for the persistence of a broad suite of seamount species.

From a conservation perspective, the magnitude of difference in effective population size between the two coral species must also be considered. Maintaining large effective size of populations is considered paramount in conservation, as small populations will be susceptible to the effects of drift and concomitant loss of genetic diversity and adaptive capacity^70^. Here we have shown that *S. variabilis* has relatively small effective population sizes compared with *D. dianthus* both in evolutionary and ecological timescales, hence *S. variabilis* populations may be particularly vulnerable to the effects of genetic drift, and this may be exacerbated if populations are further reduced e.g. from fishing or climate change. Furthermore, the low apparent migration rate and importance of asexual reproduction in *S. variabilis* are also life history traits that emphasise the vulnerability of this species^71^ relative to the sympatric *D. dianthus*.

This study provides another example of the value of genetic data to underpin informed management and conservation measures, but this is particularly so in ecosystems such as the deep sea which is remote and where it is logistically difficult to obtain ecological data in alternate ways.

## Methods

### Study species and sample collection

**Figure 1:**
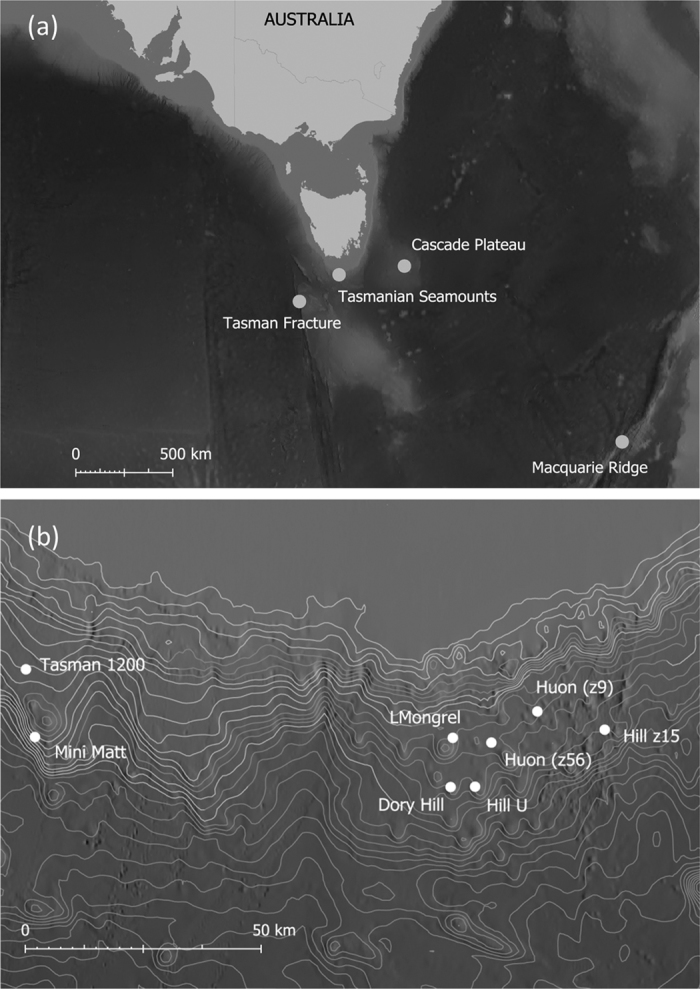
Map of four sampling regions (**a**) for the two deep sea coral species *Desmophyllum dianthus* and *Solenosmilia variabilis* in the Southern Ocean and details of individual seamounts (**b**) sampled within the Tasmanian Seamounts complex, noting five replicate sites were sampled on each of Mini Matt and Hill U seamounts. Maps were generated using Manifold GIS (V8.29) www.manifold.net based on the publically available Australia Bathymetry and Topography Grid, June 2009 obtained from Geoscience Australia (www.ga.gov.au).

**Figure 2:**
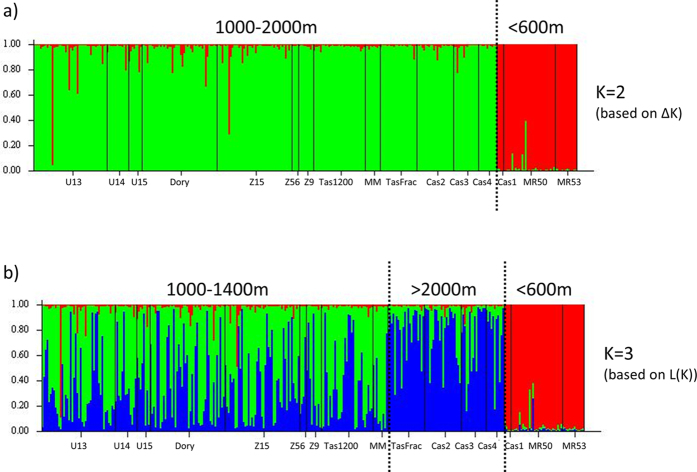
Genetic groups within *Desmophyllum dianthus*, based on output from STRUCTURE analysis and grouped by collection depth.

**Figure 3:**
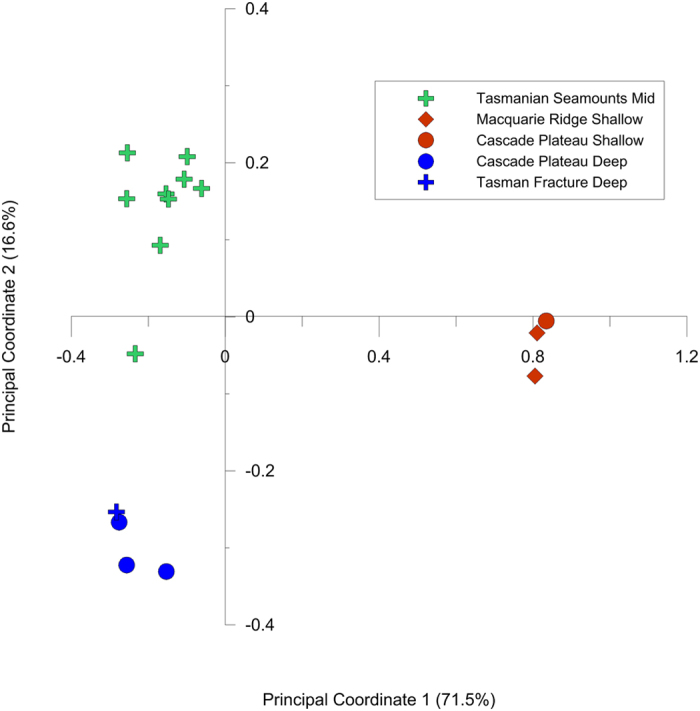
Principal coordinates analysis showing three clear groups associated with collection depth within the *Desmophyllum dianthus* samples.

**Figure 4:**
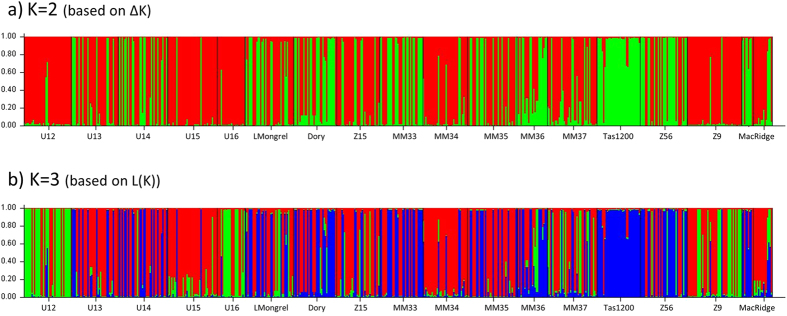
Genetic groups within *Solenosmilia variabilis* based on output from STRUCTURE analysis and grouped by seamount sampling location.

**Figure 5:**
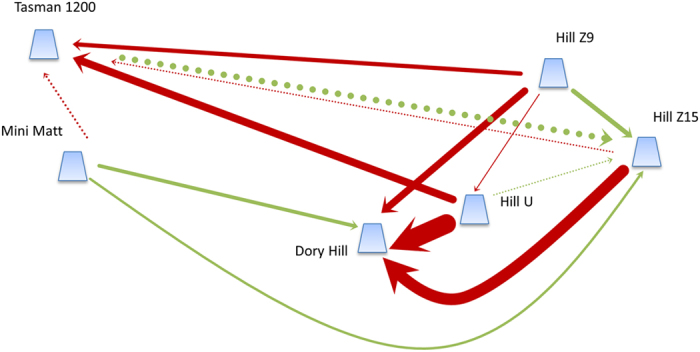
Schematic of gene flow among Tasmanian seamounts for *Desmophyllum dianthus*. Arrows indicate where there is statistically significant directional gene flow between pairs of seamounts, with the arrow indicating the direction of the highest gene flow. The width of lines is directly proportional to the magnitude of gene flow, which ranges from N_e_m = 6.96 (Hill Z9 to Hill U) to N_e_m = 151.5 (Hill U to Dory Hill). Dashed lines show other instances where N_e_m >10 (i.e. sufficient to be considered ecologically connected). Red arrows indicate westerly flow, green arrows indicate easterly flow. The figure was created using Microsoft PowerPoint based on migration rates calculated in LAMARC.

**Figure 6:**
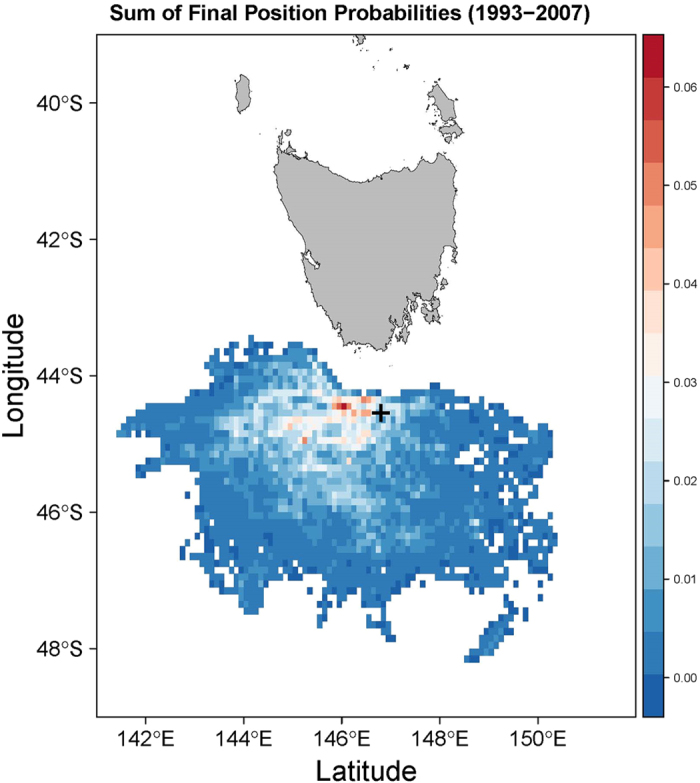
Predicted dispersal of passive particles from the Tasmanian seamounts. The dispersal probabilities were generated using “Connie2.0” CSIRO Connectivity Interface http://www.csiro.au/Connie2/ and are based on the sum across years 1993–2007. Dispersal was modelled at 995 m depth (the black cross marks release point), with a dispersal period of 25 days, and across the likely reproductive period for *D. dianthus* and *S. variabilis* in the Southern Ocean (January to May^44^). The figure was created in R version 3.1.2^89^ using the packages raster^90^, rasterVis^91^ and sp^92^.

Specimens of both species were collected by benthic sled from seamount and shelf locations at depths ranging from 489–2395 m during three research voyages in the Southern Ocean: The South East MPA Survey south of Tasmania on the RV Southern Surveyor in March/April 2007 (Voyage SS200702), the Macquarie Ridge on the RV Tangaroa in 2008 (Voyage TAN0803) and the Tasmanian Seamounts on the Thomas T. Thompson in 2008 (Voyage TT200801; samples collected by sled, and also using the Jason ROV). Sleds typically covered a few hundred metres of ground at each site. All samples were preserved whole in 95% ethanol or ultra-frozen for genetic analysis. In total 10 different seamounts and one slope location were sampled for *D. dianthus* and eight seamounts and one slope location for *S. variabilis* (Fig. 1) spanning a range of spatial scales from ~100 m to >2000 km. For both species, seamounts were classified as either “unfished” (i.e. those that were located in the Tasman Seamounts Reserve that had been closed to fishing since 1997) or “fished” (i.e. fishing activities were still permitted at the time of sampling).

### Microsatellite genotyping

The development of microsatellite loci for the two deep sea coral species is described in the Supplementary Material. DNA was extracted from a total of 326 *D. dianthus* individuals from 16 sites (Table S1) using the Qiagen DNeasy Blood and Tissue DNA Extraction Kit. PCR amplification of eight microsatellite loci was done as two separate multiplexes because allele sizes overlapped in many loci; Panel 1 - *DdB4, DdB9, DdC102, DdD109* and Panel 2 - *DdB114, DdB118, DdC6, DdC107*. The forward primer for each locus was 5′ labelled with either 6FAM, VIC, NED or PET fluorescent dyes (Table S1). Multiplex amplifications were performed in 25 μL volumes comprising 12.5 μL of 2× Qiagen Multiplex PCR Master Mix, 0.1 μM of each primer, 2.5 μL of 5× Q-solution and 10 ng DNA. Thermal cycling comprised 15 min at 95 °C, followed by 10 cycles of 94 °C for 30 sec, 59 °C annealing for 90 sec and 72 °C extension for 60 sec, then 25 cycles of 94 °C for 30 sec, 57 °C annealing for 90 sec and 72 °C extension for 60 sec with a final 30 min extension at 72 °C.

For *S. variabilis*, DNA was extracted from a total of 589 samples from 18 sites using the Qiagen DNeasy Blood and Tissue DNA Extraction Kit. Three PCR reactions were performed to amplify the nine loci (Table S2); two multiplexes (Panel 1- Sv*B106, SvC107, SvD5, SvB1*; Panel 2 - Sv*B116, SvD103, SvB123, SvD213*) and Locus *SvC4* amplified in a separate reaction. Amplifications were in 25 μL volumes comprising 12.5 μL of 2× Qiagen Multiplex PCR Master Mix, 0.1 μM of all primers excepting B116-F, B116-R, D213-F and D213-R which were added to a concentration of 0.2 μM, 2.5 μL of 5× Q-solution and 10ng DNA. Thermal cycling comprised 15 min at 95 °C, followed by 10 cycles of 94 °C for 30 sec, 57 °C annealing for 90 sec and 72 °C extension for 60 sec, then 25 cycles of 94 °C for 30 sec, 55 °C annealing for 90 sec and 72 °C extension for 60 sec with a final 30 min extension at 72 °C.

For both species, PCR products were diluted 10–20 fold into a 20 μL volume containing 9.8 μL HiDi Formamide and 0.2 μL GeneScan 500 LIZ (ABI) internal size standard, prior to electrophoresis on an ABI 3730. Alleles were analysed and scored based on fragment size using the GeneMapper V4 software (Applied Biosystems Inc.) and binned using Tandem V 1.02^72^.

### Genetic data analysis

The final data sets for each species were checked for evidence of linkage disequilibrium using Genepop 4.2^73^,^74^ and because no evidence of linkage was detected in either species, all loci were retained for further analysis. We assessed levels of intra-specific genetic variation by calculating allelic and genetic diversity in Genalex 6.5^75^, and compared the observed levels of heterozygosity within each sampling location against those expected under conditions of Hardy Weinberg Equilibrium (HWE) using Exact Tests in Genepop 4.2, and with p-values estimated using the Markov chain method based on 10,000 iterations. Significance levels were adjusted using a Bonferroni correction to account for multiple tests. Wright’s inbreeding coefficient (calculated as Weir and Cockerham’s *F*_*IS*_ in Genepop 4.2) was used to examine the size and nature of any departures from random mating. We calculated allelic richness using FSTAT^76^ for both species, and used ANOVA to compare allelic richness and expected heterozygosity between sites on fished and unfished seamounts to test the hypothesis that mean diversity differs between fished and unfished seamounts. We also calculated genotypic richness to assess the contribution of asexual reproduction to population structure as *R* = (G − 1)/(N − 1)^77^.

We tested for the presence of null alleles using Micro-checker 2.2.3^78^. Null alleles were evident in some populations at all loci for *D. dianthus* except locus *DdC107* and at two loci (Sv*B116* and Sv*D213*) in *S. variabilis*. We therefore adjusted each data set to account for null alleles using Micro-checker, based on the Oosterhout correction algorithm. Null alleles at each locus were labelled as the largest allele + one repeat. Because these adjustments are based on assumptions of HWE, which may not be appropriate for coral populations as they often reproduce asexually and can be inbred, the population genetic analyses were done on the raw data as well as the data adjusted for the presence of null alleles, for comparison.

We used STRUCTURE 2.3.3^79^ to infer the number of distinct genetic groups within the data set for both species. For each potential value of K, 20 replicate runs (including a 50,000 run burn- in and 1,000,000 MCMC replicates, using the admixture model and correlated allele frequencies) were done for the likely range of K for each species (K = 1–16 for *D. dianthus* and K = 1–17 for *S. variabilis*). Structure Harvester^80^ was used to infer the true number of genetic groups in the data set. In addition we used principal coordinates analysis using Genalex 6.5 to visualise clustering of sites related to location (for both species) and depth (for *D. dianthus*).

Analysis of connectivity among seamounts was focussed on a single depth stratum in the Tasmanian Seamounts (mid-depth range 1000–1400 m) as this sub-set of the data represented the best level of replication for both species (Table S3). Here we were able to test for differentiation at two spatial scales both for *D. dianthus* and *S. variabilis*; among sites on the same seamount (<1 km) and among seamounts (5–106 km) using AMOVA and by calculating a range of pairwise genetic differentiation measures including *F*_*ST*_, *G*’_*ST*_ and *D*_*EST*_ (using Genalex 6.5) to provide insight on evolutionary vs. contemporary relationships^81^. Significance levels in all tests were based on 10,000 permutations of the data. Only data from sites where n > 12 were used for statistical analysis (Table S3). For connectivity analyses we condensed clonal genotypes to a single representative individual at each site so as to specifically assess gene flow mediated via sexual propagules.

To assess gene flow in both species, we calculated *N*_*e*_*m* using private alleles^82^ in Genepop 4.2 as well as from estimates of *F*_*ST*_ using the formula *N*_*e*_*m* = (1/*F*_*ST*_) − 1 (Wright 1931) as each gives a different temporal perspective i.e. private alleles reflect relatively recent gene flow and *F*_*ST*_ methods represent gene flow through longer time spans^83^. Isolation by distance was examined by plotting pairwise *F*_*ST*_ against geographic distance among all sites within the mid-depth range in the Tasmanian Seamounts. Tests for a significant relationship between genetic and geographic distance were done using a Mantel Test in Genalex 6.5. In addition we examined variation in the directionality of dispersal among mid-depth seamount sites in the Tasmanian Seamounts using LAMARC 2.1.9^84^. Because there was considerable variation in sample size among sites. and to facilitate analysis of a large, multi-locus data set, we reduced sample size (by random selection) so that replication was no greater than 20 individuals at all sites. Calculation of migration among all sites within the Tasmanian Seamounts was based on a Bayesian approach. Starting values for *Θ* and *M* were based on the average of three preliminary runs using default settings and uniform prior distributions. Final runs consisted of 1 final chain, 1,000,000 steps and a burn-in of 10,000 steps, and were run with two simultaneous searches with adaptive heating and temperatures set to 1 and 1.1. Following the final run, we plotted the curve files to confirm that a single optimum value was estimated for each parameter. We calculated *N*_*e*_*m* from the LAMARC estimates of migration by multiplying effective population size (*Θ*) by migration (*M*) among all sites. Additionally we calculated contemporary effective population size using the linkage disequilibrium method of Waples and Do (2008) using NeEstimator V2^85^.

To better understand if patterns of dispersal across the Tasmanian Seamounts at mid-depth were linked to the directionality of oceanographic currents, we tested four alternate models of gene flow among the seamounts: (1) Panmixis, (2) Westward, (3) Eastward, and (4) a full model that considered gene flow among all pairs of sites. This analysis was conducted using Migrate-N v3.6.11^86^ according to the method described by Beerli & Palczewski^87^. Runs for each model used Bayesian Inference, a long chain of 10,000,000 steps with a 10,000 step burnin and were run with four simultaneous chains using static heating and swapping at every 10^th^ interval. Bayes factors and the probability of each model were calculated according to Beerli^88^. This analysis was done only for *D. dianthus* as *S. variabilis* populations were considered effectively isolated (see results).

For *D. dianthus* it was also possible to assess migration within seamounts for deep (Cascade Plateau, >2000 m) and shallow (Maquarie Ridge, <500 m) sites. These analyses were conducted as described above for the mid-depth sites in the Tasmanian Seamounts.

## Additional Information

**How to cite this article**: Miller, K. J. and Gunasekera, R. M. A comparison of genetic connectivity in two deep sea corals to examine whether seamounts are isolated islands or stepping stones for dispersal. *Sci. Rep.* **7**, 46103; doi: 10.1038/srep46103 (2017).

**Publisher's note:** Springer Nature remains neutral with regard to jurisdictional claims in published maps and institutional affiliations.

## Supplementary Material

Supplementary Information

## Figures and Tables

**Table 1:** Genotypic diversity in *Solenosmilia variabilis*.

	N	Ng	Ng:N	R
Dory Hill	34	20	0.59	0.58
Hill U 12	37	15	0.41	0.39
Hill U 13	37	22	0.59	0.58
Hill U 14	39	22	0.56	0.55
Hill U 15	39	19	0.49	0.47
Hill U 16	22	19	0.86	0.86
L Mongrel	38	20	0.53	0.51
Mac Ridge	15	10	0.67	0.64
MiniMatt33	34	16	0.47	0.45
MiniMatt34	35	26	0.74	0.74
MiniMatt35	39	15	0.38	0.37
MiniMatt36	24	14	0.58	0.57
MiniMatt37	39	30	0.77	0.76
Tasman1200	34	22	0.65	0.64
Z15	34	23	0.68	0.67
Z56	37	9	0.24	0.22
Z9	43	22	0.51	0.50

N is the total number of samples, Ng the number of unique genotypes, R is Genotypic Richness (which varies from 0 when all individuals have identical genotypes to 1 when all individuals have unique genotypes).

**Table 2:** Results from ANOVA comparing allelic richness and heterozygosity between two coral species on fished and unfished seamounts in Tasmania.

	Allelic Richness (mean ± SE)		F	P	Expected Heterozygostiy (mean ± SE)			P
	Fished	Unfished			Fished	Unfished	F	
*D. dianthus*	3.92 (0.102)	4.03 (0.084)	0.32	0.583	0.748 (0.024)	0.770 (0.045)	0.201	0.66
*S. variabilis*	3.43 (0.097)	3.14 (0.097)	3.27	0.092	0.525 (0.014)	0.519 (0.021)	0.060	0.81

**Table 3:** Pairwise genetic differentiation between *Desmophyllum dianthus* populations at mid-depth sites on Tasmanian Seamounts.

*D. dianthus*	Hill U -13	Dory Hill	Hill Z15	Tasman 1200
Hill U -13	—	0.000	0.000	0.008
Dory Hill	0.003	—	0.004	0.005
Hill Z15	0.000	0.005*	—	0.000
Tasman1200	0.005	0.004	0.004	—

Comparisons only include sites where N > 30. *F*_*ST*_ values are below the diagonal, Jost’s D is shown above the diagonal. Significance levels based on 9999 permutations are indicated as *p < 0.05, **p < 0.01 and ***p < 0.001.

**Table 4:** Pairwise genetic differentiation between *Solenosmilia variabilis* populations at mid-depth sites on Tasmanian Seamounts.

S. variabilis	Hill U Stn 12	Hill U Stn 13	Hill U Stn 14	Hill U Stn 15	Hill U Stn 16	L Mongrel	Dory Hill	Z15	MMatt Stn33	MMatt Stn34	MMatt Stn35	MMatt Stn36	MMatt Stn37	Tasman 1200	Hill Z56	Hill Z9
Hill U 12	—	0.167***	0.221***	0.153**	0.362***	0.104**	0.198***	0.132**	0.186**	0.160***	0.160**	0.186**	0.162***	0.241***	0.204**	0.203***
Hill U 13	0.093***	—	0.053*	0.062**	0.204***	0.018	0.072**	0.021	0.055*	0.022	0.044*	0.066*	0.013	0.107***	0.078*	0.069**
Hill U 14	0.125***	0.046***	—	0.103**	0.238***	0.063*	0.037*	0.045*	0.011	0.049*	0.103**	0.018	0.061**	0.050**	0.051	0.130***
Hill U 15	0.088***	0.049***	0.058***	—	0.210***	0.078**	0.150***	0.081**	0.101**	0.072**	0.083**	0.118**	0.032*	0.182***	0.082*	0.126***
Hill U 16	0.178***	0.128***	0.144***	0.119***	—	0.181***	0.313***	0.220***	0.272***	0.196***	0.184***	0.298***	0.168***	0.346***	0.299***	0.275***
LMongrel	0.056***	0.017*	0.039***	0.046***	0.094***	—	0.094**	0.002	0.053*	0.006	0.025	0.042	0.018	0.129***	0.087*	0.086***
Dory Hill	0.117***	0.056***	0.028**	0.087***	0.186***	0.058***	—	0.047*	0.000	0.102***	0.077**	0.022	0.068**	0.000	0.018	0.154***
Z15	0.062***	0.034***	0.052***	0.059***	0.115***	0.014*	0.049***	—	0.018	0.024	0.000	0.016	0.020	0.076**	0.059*	0.048**
MiniMatt 33	0.104***	0.040**	0.017*	0.072***	0.161***	0.034**	0.006	0.036**	—	0.069**	0.062*	0.000	0.050*	0.009	0.005	0.137***
MiniMatt 34	0.076***	0.030**	0.038***	0.048***	0.099***	0.010	0.069***	0.014*	0.052***	—	0.059**	0.069**	0.023*	0.143***	0.107**	0.072***
MiniMatt 35	0.082***	0.035**	0.071***	0.054***	0.105***	0.019*	0.057***	0.016*	0.046**	0.043***	—	0.059*	0.009	0.117**	0.068	0.078**
MiniMatt 36	0.102***	0.073***	0.043**	0.066***	0.155***	0.046***	0.046**	0.036**	0.049**	0.049***	0.061***	—	0.060*	0.040*	0.055	0.099**
MiniMatt 37	0.084***	0.024**	0.049***	0.028**	0.092***	0.014*	0.052***	0.018**	0.047***	0.018**	0.015*	0.048***	—	0.107***	0.057*	0.070**
Tasman1200	0.162***	0.099***	0.049***	0.119***	0.231***	0.100***	0.000	0.085***	0.038**	0.109***	0.105***	0.067***	0.095***	—	0.028	0.194***
Hill Z56	0.117***	0.062**	0.048**	0.063**	0.173***	0.061***	0.032*	0.073***	0.020	0.079***	0.058**	0.076***	0.060***	0.058**	—	0.182***
Hill Z9	0.115***	0.067***	0.108***	0.102***	0.169***	0.060***	0.125***	0.030**	0.114***	0.048***	0.071***	0.089***	0.049***	0.171***	0.151***	—

*F*_*ST*_ values are below the diagonal, Jost’s D is shown above the diagonal. Significance levels based on 9999 permutations are indicated as *p < 0.05, **p < 0.01 and ***p < 0.001. Values bounded by dashed lines are comparisons among sites on the same seamount, all other comparisons are among seamounts.
