# Supplementary material for: A comparison of genetic connectivity in two deep sea corals to examine whether seamounts are isolated islands or stepping stones for dispersal
Source: Sci Rep. 2017 Apr 10;7:46103. doi: 10.1038/srep46103 (PMC5385499; doi:10.1038/srep46103)
Supplement: Supplementary Information [file srep46103-s1.pdf]

Supporting information: Miller & Gunasekera

A comparison of genetic connectivity in two deep sea corals to examine whether seamounts are isolated islands or stepping stones for dispersal.

Development of microsatellite loci for *Desmophyllum dianthus* and *Solenosmilia variabilis*

Genomic DNA was extracted from polyp tissue of eight ethanol preserved samples of each species, from four different southeast Australian seamounts using Qiagen DNeasy Blood & Tissue DNA extraction kit (QIAGEN Inc.). Genomic DNA partial libraries were constructed using the methods of Jones et al (2002) and enriched for CA, AAC, AAG, TAGA tri- and tetra-nucleotide repeat sequences by Genetics Identification Services (GIS Chatsworth, California). This choice of motifs was guided by the relative frequencies of motifs in other scleractinian corals (Miller& Howard 2004; Shearer & Coffroth 2004; Underwood *et al.* 2007).

For *D. dianthus*, twenty-four clones from each motif-enriched library were sequenced, yielding 87 different microsatellites. Primer pairs were designed for 37 of these loci using DesignerPCR, version 1.03 (Research Genetics, Inc.) and tested on 10 individuals to optimise PCR conditions and assess polymorphism. Eight primer pairs from tri-nucleotide and four primer pairs from tetra-nucleotide loci produced amplified products of appropriate size. Subsequent genotyping based on 159 individuals across these 12 loci revealed that four loci could not be scored unambiguously, leaving eight loci that were used in the final screening.

For *S. variabilis*, 153 clones across all motif-enriched libraries were sequenced, yielding 114 different microsatellites. Primer pairs were designed for 52 microsatellites as described above and tested against seven individuals. Twenty primer pairs amplified products of the appropriate size and these were used for further optimisation and tested on 40 individuals to determine polymorphism. Of these, twelve pairs gave promising results although final genotyping revealed one to be monomorphic, and two could not be unambiguously scored and hence seven primer pairs from tri-nucleotide and two primer pairs from tetra-nucleotide loci were used in the final screening.

References

Miller KJ, Howard CG (2004) Isolation of microsatellites from two species of scleractinian coral. *Molecular Ecology Notes* **4**, 11-13.

Shearer TL, Coffroth MA (2004) Isolation of microsatellite loci from the scleractinian corals *Montastraea cavernosa* and *Porites astreoides*. *Molecular Ecology Notes* **4**, 435-437.

Underwood JN, Smith LD, Van Oppen MJH, Gilmour JP (2007) Multiple scales of genetic connectivity in a brooding coral on isolated reefs following catastrophic bleaching. *Molecular Ecology* **16**, 771-784.

Table S1. Details of eight microsatellite loci developed for *Desmophyllum dianthus*.

Locus	Repeat motif	Number of alleles	Size range (base pairs)	Primer sequences (5' to 3', forward and then reverse)
Dd B4	GTT_{11}	22	236-317	**6FAM**-CTGCTGATAAACCGAAACGATA TGGATTTTCCCAGGATATAAGG
Dd B9	TTG_{11}	20	225-282	**PET**-TGCCATCTGAGACAACAGG CAAACCAACCGAACTAAATAGG
Dd C102	TTC_{9}	22	194-308	**NED**-GAACAAGGATGGCTAAGATTG ATGAGGTATGTCGCAAACAGT
Dd D109	$ATAG_{11}$	24	254-354	**VIC**-GGTTCCACTGTACTGAGTTACG TGGCATTACCTGTAAATTAGCT
Dd C6	GAA_{13}	31	164-278	**VIC**-TTCATAAACTCGTCTGCTGTCT CCGTGGAGGACTTGGTTA
Dd C107	CTT_{8}	15	229-274	**6FAM**-ATCCACTTTTCCTTCCAGTAG CATTCCTCTTTCATTTGTTCTC
Dd B118	GTT_{8}	23	205-295	**NED**-TTGCGTTCAATGTTGGTTC AGGCTTGTCTCAGAAAATGC
Dd B114	AAC_{7}	13	133-178	**PET**-TTGGTGGCTGTAAGATGC TCTGTTGGTATTCGGTAGAGTC

Table S2. Details of nine microsatellite loci developed for *Solenosmilia variabilis*.

Locus	Repeat sequence	Number of alleles	Size range (base pairs)	Primer sequences (5' to 3', forward and then reverse)
Sv B106	[GCT]8 [GTT]6	14	240-282	**6FAM-** AGCAAGTGATGGATTACCATAC AGCCATAATGCTGTTACTTCTC
Sv C107	GAA_{14}	20	207-300	**VIC-** GCGTCTCCTTCCCTCACA GCACCCGTTGGATTATGG
Sv D5	$TAGA_{6}$	15	174-298	**NED –** AATGCTTACAACAGGAATGC GCTGAAGTTATTACGAAGAGGA
Sv B1	$[AAC]_9$ AAT $[AAC]_9$	34	119-212	**PET-** TTGAGCCTCAGATACAACTTTC GAAGCTGGCACTATTTCTGTAG
Sv B116	GTT_{12}	12	224-257	**6FAM-** GGTAATCGAGCCTACCTAACTG CGTTTGAACGAGCTTATTTTAG
Sv D103	$[TTC]_7$ TAC $[TTC]_3$	6	205-220	**VIC-** GCTTTAGGCAAAGTGTCCAC GGGCTCAACTGTAATAACAGC
Sv B123	GTT_{12}	6	165-192	**NED-** GGAGCAAGCACTCGTGTAAAT ACCAGGTAACTTTTGGCTGTG
Sv D213	$TAGA_{22}$	27	101-221	**PET-** TCAGATTCAATTCTGTTCACAC CAGGCTATAAGGCTTGACAC
Sv C4	CTT_{7}	3	203-212	**NED –**TTCCCAATTTGACTCTAAACAC TTGCTGAGAAGAAAACGTAGAT

Figure S1. Results of estimates of true K from STRUCTURE analysis of *Desmophyllum dianthus* at mid-depths in the Tasmanian Seamounts.

a)

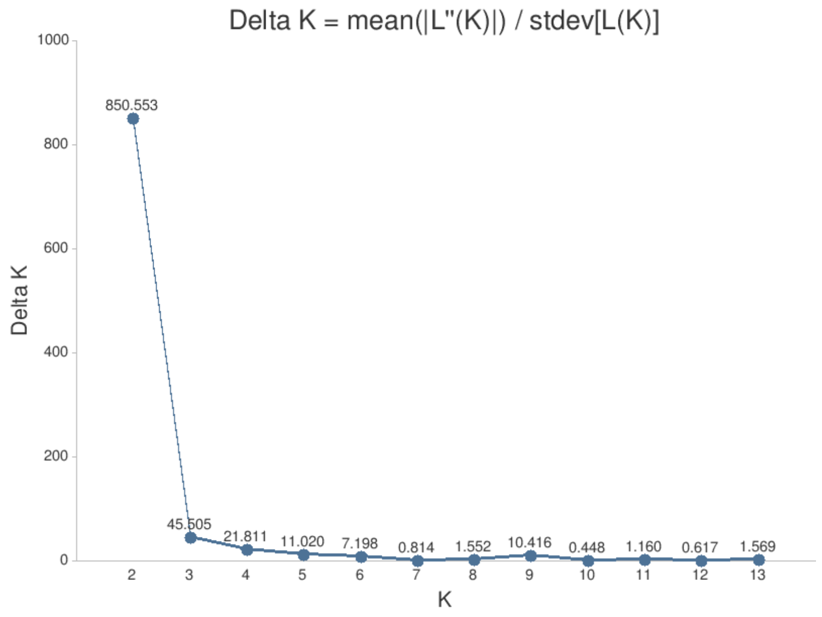

b)

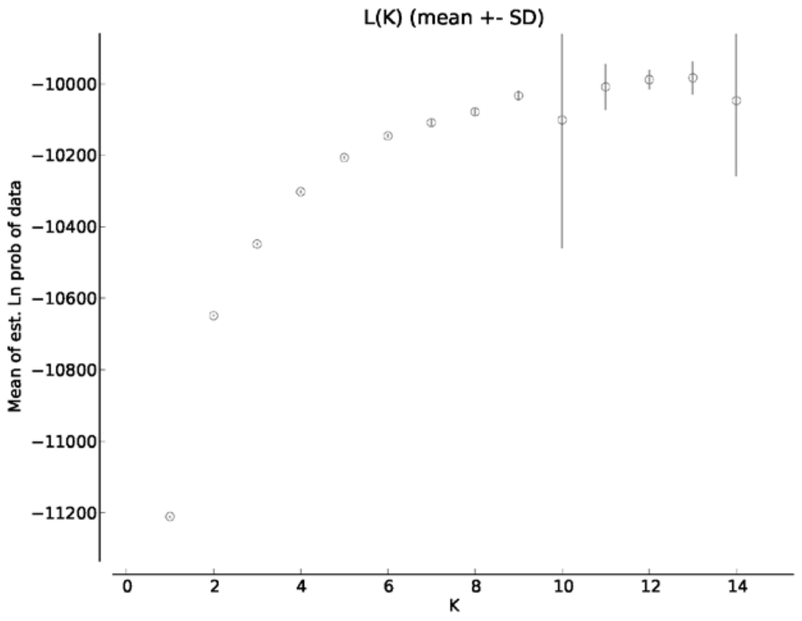

Figure S2. Results of estimates of true K from STRUCTURE analysis of *Solenosmilia variabilis* at mid-depths in the Tasmanian Seamounts.

a)

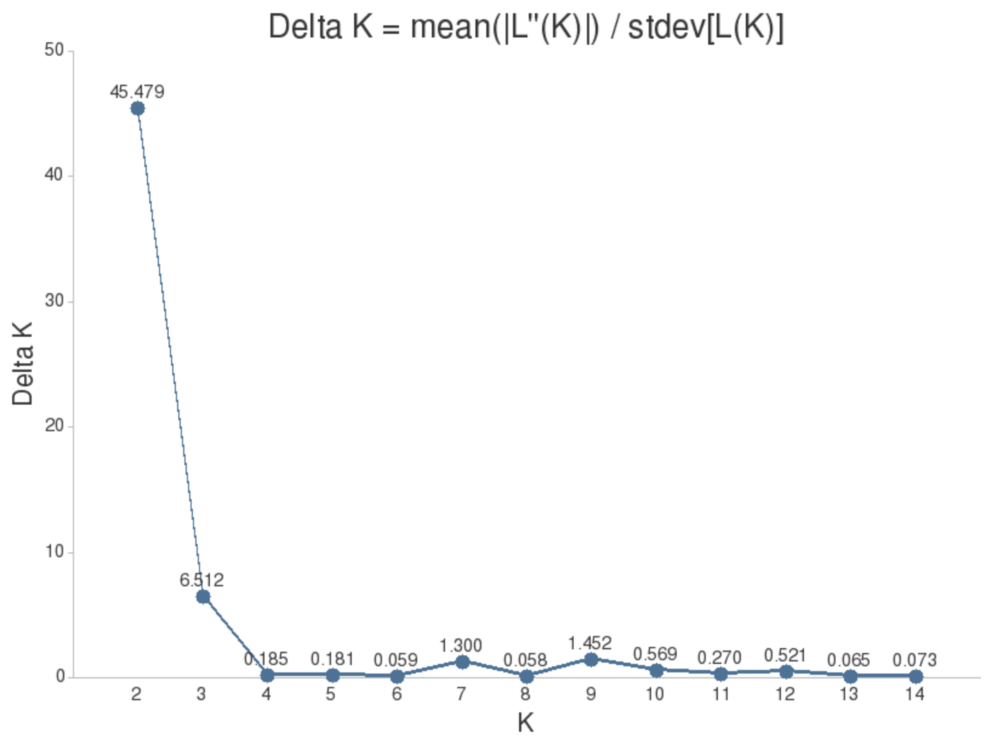

b)

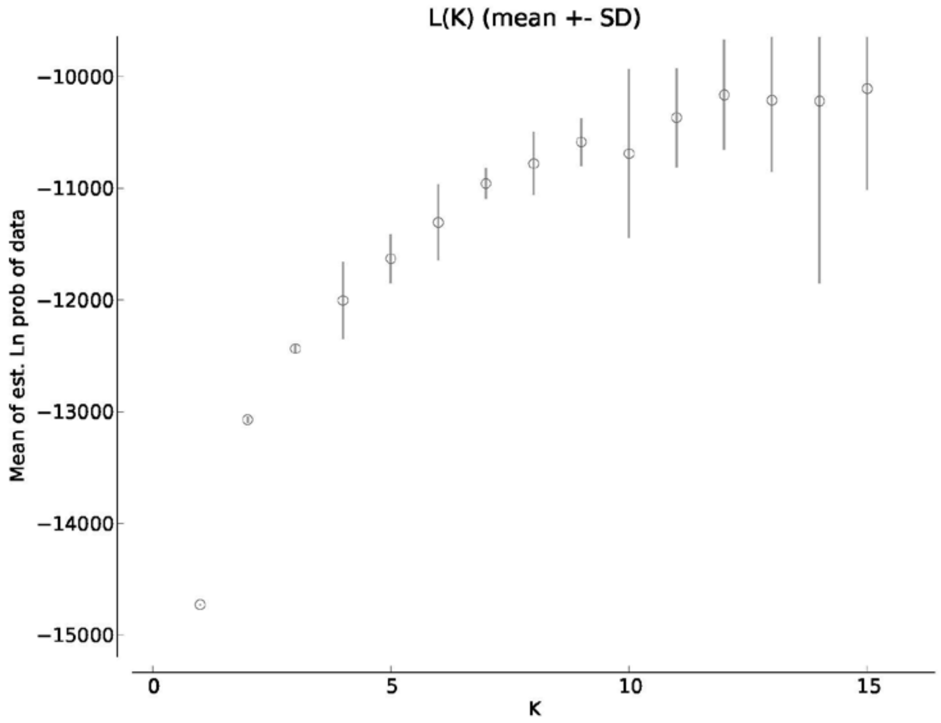

Figure S3. Particle dispersal models at 995m for the Tasmanian Seamounts for two different seasons; Summer and Winter. The dispersal probabilities were generated using “Connie2.0” CSIRO Connectivity Interface http://www.csiro.au/Connie2/ and are based on the years 2000-2001.

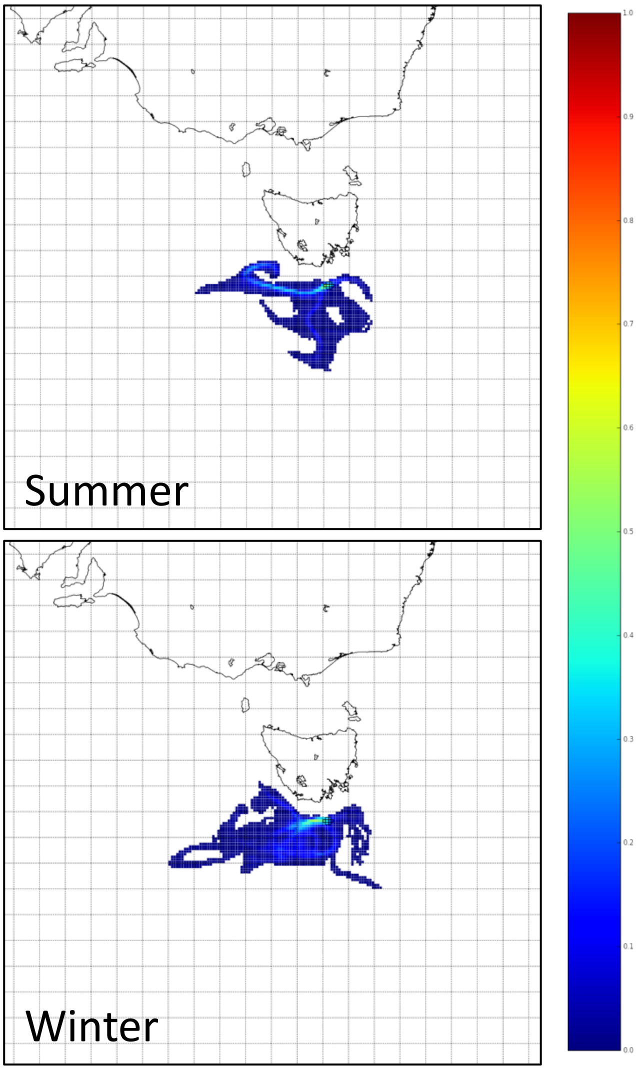

Supplementary Table S3. Details of collections of *Desmophyllum dianthus* and *Solenosmilia variabilis* from the Southern Ocean.

Operation	Region	Site/Seamount	Max Depth(m)	Latitude	Longitude	n
Desmophyllum dianthus						
SS200702/13	Tasmania	Hill U Stn 13	1300	-44.32693	147.17923	44
SS200702/14	Tasmania	Hill U Stn 14	1280	-44.32092	147.17799	13
SS200702/15	Tasmania	Hill U Stn 15	1200	-44.32245	147.18085	8
SS200702/25	Tasmania	Dory Hill	1200	-44.32626	147.11893	45
SS200702/26	Tasmania	Hill z15	1350	-44.23179	147.47266	45
SS200702/52	Tasmania	Huon (z56)	1270	-44.25307	147.21258	4
SS200702/58	Tasmania	Huon (z9)	1100	-44.20169	147.31808	9
SS200702/40	Tasmania	Tasman 1200	1180	-44.13220	146.14415	31
SS200702/34	Tasmania	Mini Matt	1310	-44.24388	146.16476	9
TT200801/J2-391-25	Tasmania	Tasman Fracture	2386	-45.36629	144.62459	22
SS200702/75	Cascade Plateau	Cascade1	660	-43.92231	150.46517	4
TT200801/J2-390-2	Cascade Plateau	Cascade2	2395	-43.80316	150.32088	22
TT200801/J2-390-4	Cascade Plateau	Cascade3	2279	-43.80588	150.32215	15
TT200801/J2-390-6	Cascade Plateau	Cascade4	2170	-43.80679	150.33791	11
TAN0803/50	Macquarie Ridge	Seamount 5	498	-51.06100	161.97800	31
TAN0803/53	Macquarie Ridge	Seamount 5	489	-51.04700	162.01883	13
Solenosmilia variabilis						
SS200702/12	Tasmania	Hill U Stn 12	1200	-44.32506	147.17963	37
SS200702/13	Tasmania	Hill U Stn 13	1300	-44.32693	147.17923	36
SS200702/14	Tasmania	Hill U Stn 14	1280	-44.32092	147.17799	39
SS200702/15	Tasmania	Hill U Stn 15	1200	-44.32245	147.18085	38
SS200702/16	Tasmania	Hill U Stn 16	1160	-44.32567	147.17516	23
SS200702/20	Tasmania	LMongrel	1200	-44.24522	147.12396	38
SS200702/25	Tasmania	Dory Hill	1200	-44.32626	147.11893	34
SS200702/26	Tasmania	Hill z15	1350	-44.23179	147.47266	33
SS200702/52	Tasmania	Huon (z56)	1270	-44.25307	147.21258	37
SS200702/58	Tasmania	Huon (z9)	1100	-44.20169	147.31808	42
SS200702/40	Tasmania	Tasman 1200	1180	-44.13220	146.14415	33
SS200702/33	Tasmania	Mini Matt Stn33	1360	-44.24473	146.16427	34
SS200702/34	Tasmania	Mini Matt Stn34	1310	-44.24388	146.16476	32
SS200702/35	Tasmania	Mini Matt Stn35	1360	-44.24496	146.16445	39
SS200702/36	Tasmania	Mini Matt Stn36	1350	-44.24493	146.16426	21
SS200702/37	Tasmania	Mini Matt Stn37	1380	-44.24423	146.16498	37
TAN0803/33	Macquarie Ridge	Seamount 3	1408	-50.09050	163.48216	15

Supplementary Table S4. Summary of genetic variability in *Desmophyllum dianthus* by locus for each site. N = sample size; Na = number of alleles; Ar = allelic richness (standardised for sample size); Ho= observed heterozygosity; He = expected heterozygosity; F = fixation index.

Desmophyllum dianthus		**B4**	**B9**	**C102**	**D109**	**C6**	**C107**	**B118**	**B114**	**Average across loci**
**Hill U -13**	**N**	43	44	40	41	44	44	41	43	42.5
	**Na**	13	11	9	16	16	7	15	6	11.6
	**Ar**	4.463	4.147	3.739	4.763	4.809	3.226	4.671	2.899	4.090
	**Ho**	0.488	0.818	0.225	0.488	0.636	0.636	0.415	0.349	0.507
	**He**	0.863	0.828	0.756	0.892	0.899	0.684	0.885	0.644	0.806
	**F**	0.434	0.012	0.702	0.453	0.292	0.069	0.532	0.459	0.369
**Hill U -14**	**N**	12	13	12	13	13	13	12	13	12.6
	**Na**	11	7	3	11	11	6	9	5	7.9
	**Ar**	4.608	3.867	1.891	4.73	4.742	3.489	4.459	3.14	3.866
	**Ho**	0.833	0.692	0.000	0.615	0.615	0.538	0.417	0.692	0.550
	**He**	0.847	0.775	0.292	0.867	0.867	0.713	0.840	0.648	0.731
	**F**	0.016	0.107	1.000	0.290	0.290	0.245	0.504	-0.068	0.298
**Hill U -15**	**N**	8	8	8	7	8	8	7	8	7.8
	**Na**	5	6	5	8	9	7	11	5	7.0
	**Ar**	3.493	4.077	3.644	4.901	4.692	3.9	5.396	3.009	4.139
	**Ho**	0.250	0.500	0.750	0.571	0.625	0.875	1.000	0.375	0.618
	**He**	0.727	0.789	0.742	0.857	0.836	0.742	0.888	0.609	0.774
	**F**	0.656	0.366	-0.011	0.333	0.252	-0.179	-0.126	0.385	0.210
**Dory Hill**	**N**	45	45	42	41	45	45	43	45	43.9
	**Na**	11	11	11	17	16	6	15	8	11.9
	**Ar**	4.577	4.118	3.773	4.939	4.622	3.052	4.513	2.974	4.071
	**Ho**	0.578	0.778	0.214	0.439	0.667	0.733	0.651	0.378	0.555
	**He**	0.880	0.822	0.778	0.909	0.879	0.673	0.870	0.657	0.809
	**F**	0.343	0.054	0.724	0.517	0.242	-0.090	0.251	0.425	0.308
**Hill Z15**	**N**	43	44	44	40	45	45	40	42	42.9
	**Na**	12	11	10	17	15	7	13	8	11.6
	**Ar**	4.556	4.255	3.466	4.805	4.906	3.288	4.743	2.947	4.121
	**Ho**	0.512	0.750	0.136	0.375	0.800	0.644	0.375	0.500	0.512
	**He**	0.875	0.842	0.715	0.896	0.908	0.704	0.892	0.620	0.806
	**F**	0.415	0.109	0.809	0.581	0.119	0.084	0.580	0.193	0.361
**Hill Z56**	**N**	4	4	3	4	4	4	4	4	3.9
	**Na**	5	4	1	5	5	3	4	3	3.8
	**Ar**	4.214	3.464	1	4.393	4.393	2.75	3.25	2.75	3.277
	**Ho**	1.000	0.500	0.000	0.250	0.750	0.500	0.500	0.250	0.469
	**He**	0.750	0.656	0.000	0.781	0.781	0.594	0.563	0.594	0.590
	**F**	-0.333	0.238	#N/A	0.680	0.040	0.158	0.111	0.579	0.210
**Hill Z9**	**N**	9	9	7	9	9	9	8	8	8.5
	**Na**	10	5	4	6	7	8	7	3	6.3
	**Ar**	4.849	4.849	4.849	4.849	4.849	4.849	4.849	4.849	3.865
	**Ho**	0.667	0.444	0.286	0.333	0.444	0.667	0.500	0.250	0.449
	**He**	0.858	0.710	0.612	0.772	0.840	0.833	0.813	0.477	0.739
	**F**	0.223	0.374	0.533	0.568	0.471	0.200	0.385	0.475	0.404
**Tas1200**	**N**	30	31	30	29	30	31	30	30	30.1
	**Na**	13	9	10	13	16	8	11	5	10.6
	**Ar**	4.646	4.096	3.961	4.223	4.916	3.306	4.396	2.821	4.046
	**Ho**	0.533	0.774	0.400	0.586	0.833	0.742	0.767	0.400	0.629

Desmophyllum dianthus		B4	B9	C102	D109	C6	C107	B118	B114	Average across loci
	He	0.878	0.825	0.808	0.832	0.903	0.682	0.852	0.628	0.801
	F	0.392	0.061	0.505	0.295	0.077	-0.089	0.100	0.363	0.213
**MiniMatt**	**N**	9	9	8	7	9	9	8	9	8.5
	**Na**	9	7	6	7	8	4	8	6	6.9
	**Ar**	4.716	4.215	3.858	4.447	4.672	3.29	4.821	3.916	4.242
	**Ho**	0.778	0.444	0.125	0.286	0.778	0.556	0.375	0.556	0.487
	**He**	0.852	0.802	0.758	0.816	0.852	0.710	0.859	0.778	0.803
	**F**	0.087	0.446	0.835	0.650	0.087	0.217	0.564	0.286	0.396
**Tas Fracture**	**N**	22	21	15	18	22	22	21	21	20.3
	**Na**	14	11	6	11	10	8	12	6	9.8
	**Ar**	4.926	4.067	2.827	4.573	4.56	3.525	4.732	3.128	4.042
	**Ho**	0.818	0.524	0.000	0.278	0.773	0.727	0.762	0.476	0.545
	**He**	0.899	0.804	0.533	0.860	0.868	0.725	0.882	0.658	0.779
	**F**	0.090	0.348	1.000	0.677	0.110	-0.003	0.136	0.276	0.329
**MacRidge**	**N**	29	31	30	31	30	31	31	26	29.9
	**Na**	11	15	7	1	20	4	14	8	10.0
	**Ar**	4.417	4.633	3.165	1	5.053	2.446	4.697	4.136	3.693
	**Ho**	0.310	0.710	0.433	0.000	0.567	0.419	0.613	0.192	0.406
	**He**	0.853	0.876	0.660	0.000	0.914	0.572	0.882	0.830	0.698
	**F**	0.636	0.190	0.343	#N/A	0.380	0.267	0.305	0.768	0.413
**MacRidge2**	**N**	12	13	12	13	13	13	13	11	12.5
	**Na**	7	10	8	1	14	4	12	5	7.6
	**Ar**	4.047	4.472	3.485	1	5.261	2.588	4.744	3.44	3.630
	**Ho**	0.417	0.846	0.500	0.000	0.385	0.615	0.692	0.273	0.466
	**He**	0.802	0.837	0.670	0.000	0.911	0.583	0.864	0.731	0.675
	**F**	0.481	-0.011	0.254	#N/A	0.578	-0.056	0.199	0.627	0.296
**Cascade 600**	**N**	4	4	4	4	4	4	4	4	4.0
	**Na**	4	6	3	1	6	2	5	4	3.9
	**Ar**	3.857	4.929	2.5	1	4.929	2	4.393	3.679	3.411
	**Ho**	0.000	1.000	0.250	0.000	0.500	0.500	0.750	0.500	0.438
	**He**	0.750	0.813	0.406	0.000	0.813	0.500	0.781	0.719	0.598
	**F**	1.000	-0.231	0.385	#N/A	0.385	0.000	0.040	0.304	0.269
**Cascade 2395**	**N**	21	19	14	22	22	22	22	20	20.3
	**Na**	11	10	5	10	10	7	12	5	8.8
	**Ar**	4.18	4.098	2.69	4.599	4.336	3.658	4.647	3.457	3.958
	**Ho**	0.762	0.632	0.214	0.591	0.636	0.818	0.455	0.300	0.551
	**He**	0.816	0.812	0.541	0.871	0.842	0.771	0.871	0.731	0.782
	**F**	0.067	0.222	0.604	0.321	0.244	-0.062	0.478	0.590	0.308
**Cascade 2279**	**N**	13	13	12	15	15	15	15	13	13.9
	**Na**	12	7	7	12	7	5	8	5	7.9
	**Ar**	4.925	4.293	3.881	4.843	4.024	3.752	4.293	3.114	4.141
	**Ho**	0.846	0.538	0.250	0.467	0.467	0.667	0.467	0.385	0.511
	**He**	0.885	0.831	0.774	0.880	0.804	0.784	0.833	0.645	0.805
	**F**	0.043	0.352	0.677	0.470	0.420	0.150	0.440	0.404	0.370
**Cascade 2170**	**N**	7	9	9	10	11	11	11	11	9.9
	**Na**	8	7	4	9	8	8	10	6	7.5
	**Ar**	4.791	4.28	2.867	4.778	4.033	4.406	4.489	2.987	4.079
	**Ho**	0.857	0.889	0.333	0.600	0.364	0.818	0.455	0.455	0.596
	**He**	0.847	0.815	0.574	0.865	0.789	0.835	0.831	0.566	0.765
	**F**	-0.012	-0.091	0.419	0.306	0.539	0.020	0.453	0.197	0.229

Desmophyllum dianthus		B4	B9	C102	D109	C6	C107	B118	B114	Average across loci
**Average across populations**	**N**	19.4	19.8	18.1	19.0	20.3	20.4	19.4	19.3	19.4
	**Na**	9.8	8.6	6.2	9.1	11.1	5.9	10.4	5.5	8.3
	**Ar**	4.732	4.265	3.687	4.861	4.891	3.753	4.807	3.242	4.279
	**Ho**	0.603	0.677	0.257	0.367	0.615	0.654	0.575	0.396	0.518
	**He**	0.836	0.802	0.601	0.694	0.857	0.694	0.838	0.658	0.748
	**F**	0.284	0.159	0.585	0.473	0.283	0.058	0.309	0.391	0.312

Supplementary Table S5. Summary of genetic variability in *Solenosmilia variabilis* by locus for each site. N = sample size; Na = number of alleles; Ar = allelic richness (standardised for sample size); Ho= observed heterozygosity; He = expected heterozygosity; F = fixation index.

Solenosmilia variabilis		B1	B106	B116	B123	C107	C4	D103	D213	D5	Average across loci
**Hill U Stn 12**	**N**	37	36	36	36	28	37	37	37	35	35.4
	**Na**	10	6	4	2	6	1	3	8	6	5.1
	**Ar**	4.586	3.063	2.852	1.981	3.848	1	2.086	3.437	3.453	2.923
	**Ho**	1.000	0.333	0.972	0.056	0.643	0.000	0.054	0.297	0.971	0.481
	**He**	0.746	0.498	0.602	0.424	0.726	0.000	0.388	0.517	0.651	0.506
	**F**	-0.341	0.331	-0.614	0.869	0.114	#N/A	0.861	0.425	-0.493	0.144
**Hill U Stn 13**	**N**	37	35	37	30	36	36	37	37	36	35.7
	**Na**	11	5	4	2	6	1	4	10	7	5.6
	**Ar**	4.429	2.814	3.268	1.308	4.055	1	3.314	5.412	4.081	3.298
	**Ho**	0.405	0.314	0.568	0.000	0.639	0.000	0.541	0.676	0.528	0.408
	**He**	0.721	0.407	0.671	0.064	0.736	0.000	0.617	0.817	0.671	0.523
	**F**	0.438	0.228	0.154	1.000	0.132	#N/A	0.123	0.173	0.213	0.308
**Hill U Stn 14**	**N**	37	36	36	39	34	39	39	33	37	36.7
	**Na**	6	5	6	3	5	1	4	8	7	5.0
	**Ar**	3.739	3.667	4.643	1.256	3.912	1	2.711	5.344	3.866	3.349
	**Ho**	0.514	0.583	0.833	0.051	0.441	0.000	0.615	0.455	0.270	0.418
	**He**	0.614	0.649	0.802	0.050	0.661	0.000	0.473	0.834	0.698	0.531
	**F**	0.164	0.101	-0.039	-0.020	0.333	#N/A	-0.301	0.455	0.613	0.163
**Hill U Stn 15**	**N**	37	35	37	39	38	37	38	36	35	36.9
	**Na**	9	5	5	3	6	1	3	6	5	4.8
	**Ar**	4.444	3.147	3.451	1.256	4.414	1	2.947	4.694	3.801	3.239
	**Ho**	0.405	0.714	0.405	0.051	0.947	0.000	0.579	1.000	0.657	0.529
	**He**	0.749	0.563	0.657	0.050	0.738	0.000	0.655	0.788	0.709	0.546
	**F**	0.459	-0.269	0.383	-0.020	-0.285	#N/A	0.116	-0.269	0.074	0.024
**Hill U Stn 16**	**N**	20	20	20	16	17	22	21	19	21	19.6
	**Na**	8	2	5	1	4	2	3	6	4	3.9
	**Ar**	5.243	1.25	2.748	1	3.383	1.974	2.665	4.371	3.368	2.889
	**Ho**	1.000	0.050	0.250	0.000	0.882	0.545	0.619	0.632	0.476	0.495
	**He**	0.798	0.049	0.563	0.000	0.645	0.397	0.541	0.740	0.685	0.491
	**F**	-0.254	-0.026	0.556	#N/A	-0.367	-0.375	-0.145	0.146	0.305	-0.020
**LMongrel**	**N**	37	37	37	34	36	38	38	29	37	35.9
	**Na**	11	7	5	2	7	1	4	13	7	6.3
	**Ar**	5.091	3.734	3.642	1.741	4.396	1	2.81	6.356	4.577	3.705
	**Ho**	0.297	0.622	0.189	0.000	0.556	0.000	0.684	0.552	0.730	0.403
	**He**	0.761	0.684	0.714	0.208	0.736	0.000	0.569	0.867	0.775	0.590
	**F**	0.609	0.092	0.735	1.000	0.245	#N/A	-0.203	0.364	0.058	0.363

Solenosmilia variabilis		B1	B106	B116	B123	C107	C4	D103	D213	D5	Average across loci
**Dory Hill**	**N**	31	30	33	33	33	34	33	31	32	32.2
	**Na**	7	10	4	1	6	1	3	12	7	5.7
	**Ar**	3.288	5.219	3.478	1	3.745	1	2.278	6.419	4.411	3.426
	**Ho**	0.484	0.800	0.242	0.000	0.667	0.000	0.394	0.290	0.625	0.389
	**He**	0.508	0.801	0.693	0.000	0.607	0.000	0.512	0.877	0.750	0.528
	**F**	0.048	0.001	0.650	#N/A	-0.098	#N/A	0.231	0.669	0.167	0.239
**Z15**	**N**	31	31	34	26	29	33	34	27	29	30.4
	**Na**	12	11	7	2	7	2	4	15	8	7.6
	**Ar**	5.553	3.925	3.729	1.351	5.007	1.152	2.293	6.278	4.191	3.720
	**Ho**	0.742	0.581	0.765	0.000	0.690	0.030	0.324	0.741	0.345	0.468
	**He**	0.824	0.562	0.684	0.074	0.806	0.030	0.529	0.857	0.714	0.564
	**F**	0.100	-0.033	-0.118	1.000	0.144	-0.015	0.388	0.136	0.517	0.235
**MiniMatt Stn33**	**N**	33	34	34	30	33	34	34	33	34	33.2
	**Na**	8	10	6	1	6	1	4	9	7	5.8
	**Ar**	3.437	4.583	4.203	1	3.115	1	2.85	5.473	4.464	3.347
	**Ho**	0.485	0.559	0.706	0.000	0.455	0.000	0.294	0.273	0.500	0.363
	**He**	0.478	0.712	0.740	0.000	0.541	0.000	0.517	0.829	0.770	0.510
	**F**	-0.014	0.215	0.047	#N/A	0.160	#N/A	0.431	0.671	0.351	0.266
**MiniMatt Stn34**	**N**	33	32	30	28	28	34	35	29	31	31.1
	**Na**	13	9	6	2	11	1	4	14	7	7.4
	**Ar**	5.832	3.994	4.779	1.93	5.385	1	2.871	6.827	3.927	4.061
	**Ho**	0.758	0.781	0.433	0.071	0.929	0.000	0.486	0.207	0.645	0.479
	**He**	0.819	0.612	0.799	0.337	0.800	0.000	0.511	0.888	0.683	0.606
	**F**	0.075	-0.277	0.458	0.788	-0.160	#N/A	0.050	0.767	0.056	0.220
**MiniMatt Stn35**	**N**	37	39	39	37	38	39	39	38	37	38.1
	**Na**	11	7	4	1	7	1	3	12	7	5.9
	**Ar**	4.783	4.117	3.175	1	3.679	1	2.14	4.439	3.317	3.072
	**Ho**	0.865	0.949	0.564	0.000	0.632	0.000	0.077	0.184	0.730	0.444
	**He**	0.787	0.700	0.671	0.000	0.712	0.000	0.351	0.697	0.617	0.504
	**F**	-0.099	-0.355	0.159	#N/A	0.112	#N/A	0.781	0.736	-0.184	0.164
**MiniMatt Stn36**	**N**	21	18	21	23	16	24	19	23	18	20.3
	**Na**	5	6	5	2	4	1	4	10	6	4.8
	**Ar**	2.696	4.735	3.311	1.217	2.716	1	2.698	4.386	4.187	2.994
	**Ho**	0.381	0.944	0.381	0.043	0.188	0.000	0.158	0.435	0.833	0.374
	**He**	0.336	0.790	0.593	0.043	0.408	0.000	0.494	0.609	0.744	0.446
	**F**	-0.135	-0.195	0.358	-0.022	0.541	#N/A	0.681	0.286	-0.120	0.174
**MiniMatt Stn37**	**N**	30	35	38	33	34	37	39	33	36	35.0
	**Na**	10	7	5	1	9	1	6	14	6	6.6
	**Ar**	5.017	3.826	3.58	1	4.293	1	3.367	6.557	3.702	3.594
	**Ho**	0.733	0.629	0.289	0.000	0.735	0.000	0.692	0.727	0.556	0.485
	**He**	0.755	0.642	0.683	0.000	0.724	0.000	0.657	0.882	0.698	0.560
	**F**	0.029	0.022	0.576	#N/A	-0.016	#N/A	-0.053	0.175	0.204	0.134
**Tasman1200**	**N**	32	25	33	34	33	34	33	29	30	31.4
	**Na**	4	8	6	1	7	1	4	11	5	5.2
	**Ar**	2.422	5.142	3.113	1	2.996	1	2.62	6.273	3.825	3.155
	**Ho**	0.375	0.920	0.394	0.000	0.485	0.000	0.303	0.655	0.567	0.411
	**He**	0.363	0.798	0.524	0.000	0.429	0.000	0.399	0.871	0.676	0.451
	**F**	-0.032	-0.153	0.249	#N/A	-0.129	#N/A	0.241	0.248	0.161	0.083
**Z56**	**N**	37	37	37	37	34	37	37	37	37	36.7
	**Na**	5	7	7	1	6	1	4	6	8	5.0

Solenosmilia variabilis		B1	B106	B116	B123	C107	C4	D103	D213	D5	Average across loci
	**Ar**	3.194	3.72	4.61	1	3.954	1	3.589	4.868	4.751	3.410
	**Ho**	0.405	0.595	0.541	0.000	0.618	0.000	0.541	0.270	0.703	0.408
	**He**	0.536	0.662	0.791	0.000	0.641	0.000	0.685	0.804	0.757	0.542
	**F**	0.243	0.102	0.316	#N/A	0.036	#N/A	0.211	0.664	0.071	0.235
**Z9**	**N**	39	40	43	21	36	42	43	37	41	38.0
	**Na**	7	7	4	1	8	1	3	9	5	5.0
	**Ar**	4.969	4.004	2.047	1	5.458	1	2.405	5.244	2.836	3.218
	**Ho**	0.949	0.550	0.186	0.000	0.944	0.000	0.488	0.541	0.146	0.423
	**He**	0.794	0.665	0.232	0.000	0.837	0.000	0.393	0.794	0.587	0.478
	**F**	-0.195	0.173	0.199	#N/A	-0.128	#N/A	-0.241	0.319	0.751	0.125
**MacRidge**	**N**	13	11	12	8	13	15	12	11	13	12
	**Na**	8	3	3	2	6	1	2	6	9	4.4
	**Ar**	4.586	2.424	2.949	1.875	4.513	1	1.999	4.747	5.608	3.300
	**Ho**	0.615	0.545	0.083	0.000	0.923	0.000	0.833	0.636	0.923	0.507
	**He**	0.666	0.417	0.635	0.219	0.751	0.000	0.486	0.748	0.802	0.525
	**F**	0.076	-0.307	0.869	1.000	-0.228	#N/A	-0.714	0.149	-0.151	0.087
**Average across populations**	**N**	30.6	29.9	31.4	28.5	28.9	32.3	32.1	29.2	30.2	30.3
	**Na**	8.3	6.7	5.0	1.6	6.3	1.1	3.6	9.7	6.4	5.4
	**Ar**	5.248	4.501	4.194	1.65	4.969	1.108	3.02	7.399	5.01	4.122
	**Ho**	0.591	0.609	0.461	0.015	0.676	0.032	0.439	0.485	0.600	0.434
	**He**	0.646	0.599	0.641	0.082	0.669	0.024	0.499	0.786	0.702	0.516
	**F**	0.088	-0.013	0.272	0.622	-0.004	-0.195	0.130	0.383	0.149	0.170

Supplementary Table S6. Estimates of migration among *Desmophyllum dianthus* populations from mid-depths (1000-1400m) on Tasmanian seamounts from LAMARC. Values in bold represent instances where there was significant asymmetrical gene flow between pairs of sites ($p<0.05$). Values indicated as * have $N_e m$ significantly >1 and values marked with † have $N_e m$ significantly <1 based on the 95% CI ($N_e m$ = migration rate x Θ of recipient population).

	Migration to					
Migration from	Hill U	Dory Hill	Hill Z15	Hill Z9	Tasman 1200	Mini Matt
Hill U	-	**16.366***	1.321*	**0.096**	**6.060***	0.122*
Dory Hill	**0.744***	-	**0.741***	**0.012†**	0.795*	**0.030**
Hill Z15	0.412*	**9.594***	-	**0.119**	1.186*	**0.062**
Hill Z9	**0.700***	**5.871***	**3.667***	-	**3.354***	0.303*
Tasman 1200	**0.099**	5.414*	0.835*	**0.016**	-	0.205*
Mini Matt	0.923*	**2.933***	**1.962***	0.067	1.819*	-

Supplementary Table S7. Estimates of migration among *Solenosmilia variabilis* populations on Tasmanian seamounts from LAMARC. Values in bold represent instances where there was significant asymmetrical gene flow between pairs of sites ($p<0.05$). Values indicated as * have $N_e m$ significantly >1 and values marked with † have $N_e m$ significantly <1 based on the 95% CI ($N_e m$ = migration rate x theta of recipient population).

	Migration to							
Migration from	Hill U	LMongrel	Dory Hill	Hill Z15	Mini Matt	Tasman 1200	Hill Z56	Hill Z9
Hill U	-	0.017†	0.503†	0.203	5.817	1.297†	**0.209***	5.202†
LMongrel	8.146	-	4.633†	**8.026**	**7.453**	1.647†	0.109	**4.431†**
Dory Hill	7.684†	0.216†	-	4.541	3.765	0.302	0.061	8.960
Hill Z15	6.479†	**0.026†**	0.242†	-	4.889	0.029	**0.050**	**13.432**
Mini Matt	23.478†	**0.011†**	1.324†	16.672*	-	**0.052†**	**0.289***	9.923
Tasman 1200	5.823†	0.304†	2.144	7.028	**5.080**	-	0.056	4.292
Hill Z56	**7.143**	0.458†	0.865	**91.350***	**91.466***	1.859†	-	**42.121**
Hill Z9	5.809	**0.032†**	0.402†	**8.583**	12.146	0.221	**0.077**	

Supplementary Table S8. Estimates of gene flow among *Desmophyllum dianthus* populations on individual seamounts as migration (*m*) and number of effective migrants per generation ($N_e m$) from LAMARC; values for each pairwise estimate are listed as $m/N_e m$. Values in bold represent instances where there was significant asymmetrical gene flow between pairs of sites ($p<0.05$). Values indicated as * have $N_e m$ significantly >1 based on the 95% confidence interval ($N_e m$ = migration rate x theta of recipient population). No estimates of $N_e m$ were significantly <1. Note that small sample sizes at some sites may limit inference of connectivity from these results.

Migration from	n	Migration to		
**Tasmania**		Hill U 13	Hill U 14	Hill U 15
Hill U 13	44	-	0.432/4.15	0.650/6.48*
Hill U 14	13	0.701/6.98*	-	**0.494/4.92***
Hill U 15	8	0.674/6.71*	**4.705/45.21***	-
**Cascade Plateau**		Cascade 2170	Cascade 2279	Cascade 2395
Cascade 2170	22	-	2.688/25.14*	0.360/3.02
Cascade 2279	15	3.320/31.8*	-	3.783/31.8
Cascade 2395	11	2.703/25.89*	1.594/14.91*	-
**Macquarie Ridge**		Seamount 5 Stn50	Seamount 5 Stn53	
Seamount 5 Stn50	31	-	4.719/45.69*	
Seamount 5 Stn53	13	4.864/46.84*	-	

Supplementary Table S9. Estimates of gene flow among *Solenosmilia variabilis* populations on individual seamounts as migration (*m*; upper value) and number of effective migrants per generation (N_em; lower value in parentheses) from LAMARC. Values in bold represent instances where there was significant asymmetrical gene flow between pairs of sites (p<0.05). Values indicated as * have N_em significantly >1 and values marked with † have N_em significantly <1 based on the 95% CI (N_em = migration rate x theta of recipient population).

Migration from	Migration to Hill U 12	Hill U 13	Hill U 14	Hill U 15	Hill U 16	Mini Matt 33	Mini Matt 34	Mini Matt 35	Mini Matt 36	Mini Matt 37
Hill U 12	-	0.286<br>(2.8*)	**3.878<br>(0.2†)**	**63.010<br>(4.0*)**	**91.699<br>(1.4*)**					
Hill U 13	0.340<br>(3.3*)	-	**4.517<br>(0.1†)**	**4.561<br>(0.3†)**	**6.185<br>(0.1†)**					
Hill U 14	**0.301<br>(2.9*)**	**0.138<br>(1.3)**	-	1.136<br>(0.1†)	5.148<br>(0.1†)					
Hill U 15	**0.616<br>(6.0*)**	**0.149<br>(1.4*)**	4.924<br>(0.2)	-	6.412<br>(0.1†)					
Hill U 16	**0.193<br>(1.9*)**	**0.148<br>(1.4)**	1.900<br>(0.1)	20.253<br>(1.3)	-					
Mini Matt 33						-	0.258<br>(2.5*)	0.701<br>(0.1†)	0.222<br>(2.1*)	0.869<br>(3.9)
Mini Matt 34						0.199<br>(0.6)	-	0.317<br>(0.03)	0.742<br>(6.9*)	2.115<br>(9.6*)
Mini Matt 35						0.271<br>(0.8)	0.377<br>(3.7*)	-	**0.107<br>(1.0)**	0.210<br>(1.0)
Mini Matt 36						0.262<br>(0.8)	0.447<br>(4.4*)	**1.036<br>(0.1)**	-	0.302<br>(1.4)
Mini Matt 37						0.620<br>(1.9)	0.275<br>(2.7*)	1.366<br>(0.2)	0.456<br>(4.3*)	-
